# Transcriptome analysis of the winter wheat *Dn1* in response to cold stress

**DOI:** 10.1186/s12870-022-03654-1

**Published:** 2022-06-06

**Authors:** Yu Tian, Kankan Peng, Guicheng Lou, Zhipeng Ren, Xianze Sun, Zhengwei Wang, Jinpu Xing, Chunhua Song, Jing Cang

**Affiliations:** College of Life Science, Northeast Agricultural University, Harbin, 150030 People’s Republic of China

**Keywords:** *Dongnongdongmai1*, Cold stress, RNA-seq, Cold signal transduction, Transcription factor, Carbohydrate metabolism

## Abstract

**Background:**

Heilongjiang Province has a long and cold winter season (the minimum temperature can reach -30 ℃), and few winter wheat varieties can safely overwinter. *Dongnongdongmai1* (*Dn1*) is the first winter wheat variety that can safely overwinter in Heilongjiang Province. This variety fills the gap for winter wheat cultivation in the frigid region of China and greatly increases the land utilization rate. To understand the molecular mechanism of the cold response, we conducted RNA-sequencing analysis of *Dn1* under cold stress.

**Results:**

Approximately 120,000 genes were detected in *Dn1* under cold stress. The numbers of differentially expressed genes (DEGs) in the six comparison groups (0 ℃ vs. 5 ℃, -5 ℃ vs. 5 ℃, -10 ℃ vs. 5 ℃, -15 ℃ vs. 5 ℃, -20 ℃ vs. 5 ℃ and -25 ℃ vs. 5 ℃) were 11,313, 8313, 15,636, 13,671, 14,294 and 13,979, respectively. Gene Ontology functional annotation suggested that the DEGs under cold stress mainly had “binding”, “protein kinase” and “catalytic” activities and were involved in “oxidation–reduction”, “protein phosphorylation” and “carbohydrate metabolic” processes. Kyoto Encyclopedia of Genes and Genomes enrichment analysis indicated that the DEGs performed important functions in cold signal transduction and carbohydrate metabolism. In addition, major transcription factors (AP2/ERF, bZIP, NAC, WRKY, bHLH and MYB) participating in the *Dn1* cold stress response were activated by low temperature.

**Conclusion:**

This is the first study to explore the *Dn1* transcriptome under cold stress. Our study comprehensively analysed the key genes involved in cold signal transduction and carbohydrate metabolism in *Dn1* under cold stress. The results obtained by transcriptome analysis could help to further explore the cold resistance mechanism of *Dn1* and provide basis for breeding of cold-resistant crops.

**Supplementary Information:**

The online version contains supplementary material available at 10.1186/s12870-022-03654-1.

## Highlights

• Approximately 120,000 genes were detected in Dn1 during the overwintering stage (the lowest sampling temperature was -25 °C), of which 31,000 genes were differentially expressed.

• A total of 1230 differentially expressed genes involved in cold signal perception and transduction were detected in Dn1 during the overwintering stage.

• A total of 1715 differentially expressed genes involved in carbohydrate metabolism were detected in Dn1 during the overwintering stage, most of which were enriched in glycolysis/gluconeogenesis and starch and sucrose metabolism.

**Supplementary Information:**

The online version contains supplementary material available at 10.1186/s12870-022-03654-1.

## Background

Cold stress, a major type of abiotic stress, is able to restrict the growth, development, yield and geographic distribution of many important crops [1]. Cold stress is divided into chilling stress (0–15 ℃) and freezing stress (< 0 ℃), which can cause different degrees of damage to plants [2]. Chilling stress can lead to hardening of the cell membrane, instability of protein complexes, and disruption of photosynthesis. Freezing stress promotes the formation of ice in the intercellular space, and the accumulated intercellular ice disrupts the cell membrane [3]. Wheat (*Triticum aestivum* L.) is one of the most important food crops in China [4]. In winter, it is extremely cold in northeast China, especially Heilongjiang Province where the minimum temperature can reach -30 ℃. The low temperature and insufficient effective accumulated temperature cause the cultivated wheat to ripen once a year, with a low land utilization rate, which is the main reason for the lower wheat cultivation area in Heilongjiang Province. Therefore, exploring the cold resistance mechanism of wheat and enhancing the cold resistance of wheat are of great significance to agricultural development in alpine regions.

Plants have evolved complex physiological and molecular mechanisms to withstand cold stress. One of the best characterized mechanisms is cold acclimation [5]. During this process, a series of physiological and biochemical changes occur. Plants synthesize proline, soluble sugar and protective proteins (late embryogensis abundant proteins, antifreeze proteins and cold shock proteins) to increase their cold stress tolerance [6]. Among the numerous and complex cold acclimation signalling pathways, the best understood ICE-CBF-COR pathway, initiated by the calcium signalling cascade, plays a central role [7]. In recent years, researchers have also found that CBF-dependent pathways are modulated by important regulators at the transcriptional, posttranscriptional and posttranslational levels [8]. RNA-sequencing (RNA-seq) is a powerful technology capable of revealing the global transcriptional activities of any species at the mononucleotide level [9, 10].

In previous studies, researchers have comprehensively analysed gene functions and metabolic pathways at the transcriptional level under cold stress using RNA-seq technology [9, 11, 12]. Recent plant transcriptome studies have found that calcium ion (Ca^2+^) and reactive oxygen species (ROS) mediated signalling pathways and mitogen activated protein kinase (MAPK) cascades in plants are able to rapidly respond to cold stress [13, 14]. The cold response of plants is a complex process that involves the regulation of a set of transcription factors (TFs) and various genes. Jiang et al. [15] revealed the complex mechanism of TFs in peanut (*Arachis hypogaea* L.) in response to cold stress and the importance of hormone signal transduction and plant-pathogen interactions in plant cold tolerance using RNA-seq. Zhou et al. [11] found that the expression of some members of the AP2/ERF, WRKY, NAC, and bZIP families of jujube (*Ziziphus jujuba* Mill.) was significantly up/downregulated under freezing stress and that galactose metabolism was involved in plant freezing tolerance using RNA-seq. Dong et al. [9] found significant changes in related gene expression in glycolysis and the TCA cycle in the *Poa pratensis* L. transcriptome, which are involved in improving cold tolerance in plants.

In recent years, there have been several reports on the transcriptome of wheat under drought [16, 17], cold [18–20], heat [21] and water [22] stress. All three studies of the wheat transcriptome under cold stress were conducted on seedling-stage wheat cultivated in a greenhouse. Díaz  [19] and Aleliūnas [20] sampled winter wheat leaves under chilling stress at 2 ℃ and 5 ℃ for transcriptome sequencing, respectively, and found that hormone signalling pathways, lipid and carbohydrate metabolism, and amino acid accumulation were involved in plant cold tolerance. Zhao et al. [18] comprehensively analysed the transcriptome and metabolome of winter wheat crowns under chilling and freezing stress and found that abscisic acid/jasmonic acid signalling and proline biosynthesis played important roles in regulating wheat cold tolerance. However, transcriptome study of tillering nodes of winter wheat in the field under cold stress is still scarce.

Winter wheat is sown and cultivated in autumn and harvested in the following summer, and one aim is to utilize winter moisture to increase yield [23]. *Dongnongdongmai1* (*Dn1*) is the first wheat variety that is capable of safe overwintering in the frigid region of Heilongjiang Province, and the regreening rate after winter is greater than 85%. Our previous studies found that *Dn1* transcription factor (TabZIP1) [24] and key carbohydrate metabolism enzymes (TaG6PDH, Ta6PGDH and TaFBA-A10) [25, 26] are involved in improving plant cold tolerance. We also demonstrated the important role of tillering nodes in the wintering stage of winter wheat [25, 26]. The adaptation of plants to low temperature is a complex process that requires the joint regulation of multiple biological processes. To respond to these biological processes, plants need to alter the expression of a large number of genes. Therefore, it is difficult to reveal the complex mechanism underlying the cold response by studying a single gene and a single metabolic pathway. In this study, we comprehensively analysed the transcriptome of winter wheat *Dn1* in the field under cold stress, aiming to discover important cold resistance genes and explore the complex cold resistance mechanisms activated during the wintering period.

## Results

### Changes in *Dn1* phenotype at low temperatures

**Fig. 1:**
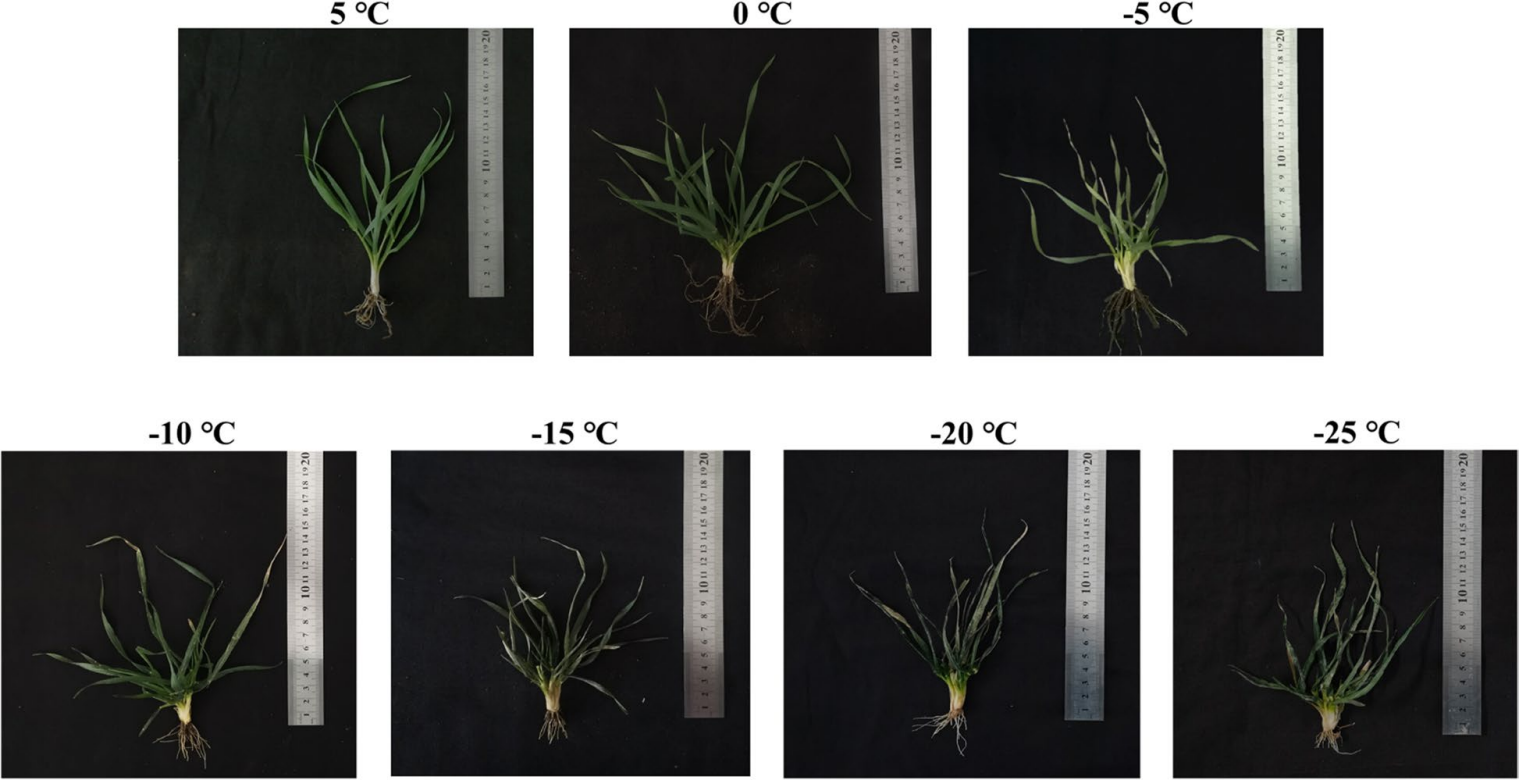
Dn1 phenotype at low temperatures

Our results showed the changes in *Dn1* phenotype under cold stress. Under chilling temperatures (5 ℃ and 0 ℃), *Dn1* leaves grew normally. When the temperature dropped below 0 ℃, the leaves lost water and wilted. However, tillering nodes consistently maintained good growth under cold stress (Fig. 1).

### Quality analysis and sequence assembly of RNA-seq data

The winter wheat tillering nodes were collected at 5 ℃, 0 ℃, -5 ℃, -10 ℃, -15 ℃, -20 ℃ and -25 ℃ (average minimum temperature for ten consecutive days). Total RNA extracted from tillering nodes by using the Illumina Hiseq 4000 sequencing system was used to construct an expression library for sequencing. We obtained 21 library reads (three biological replicates for each temperature sample), and each library had approximately 49 million reads. There were approximately 45 million clean reads per library after filtering, which represented on average 91.23% of the total reads of all libraries. In all samples, the Q30 value was over 98%, and the guanine and cytosine (GC) content was 49%. There are approximately 42 million reads per library that could be aligned to the genome, of which there are approximately 31 million reads that could only be uniquely aligned to one location on the genome and 11 million reads that could be aligned to multiple locations on the genome (Table 1). A total of approximately 120,000 genes were detected in each library (Table S1). This suggests that the RNA-seq data are robust quality and reliable results are obtained from the transcriptome assembly.

**Table 1:** Summary of Dn1 RNA-seq data

Sample	Raw Data	Valid Data	Valid Ratio	Q30%	GC content%	Mapped reads	Unique Mapped reads	Multi Mapped reads
Tn_5_1	47,236,140	43,092,004	91.23	98.62	50	41,054,960	30,160,876	10,894,084
Tn_5_2	40,096,482	31,285,112	78.02	98.58	50	29,635,962	22,139,512	7,496,450
Tn_5_3	44,923,136	37,885,726	84.33	98.55	50	35,992,455	26,603,295	9,389,160
Tn_0_1	47,686,166	45,009,446	94.39	98.62	50	42,851,440	31,751,724	11,099,716
Tn_0_2	49,153,908	45,206,694	91.97	98.49	49	42,977,551	315,54,028	11,423,523
Tn_0_3	54,010,602	47,469,770	87.89	98.46	50	45,037,829	33,019,695	12,018,134
Tn_M5_1	55,596,548	51,055,942	91.83	98.58	49	48,440,883	35,325,780	13,115,103
Tn_M5_2	51,441,478	49,053,588	95.36	98.78	49	46,877,584	35,058,768	11,818,816
Tn_M5_3	53,207,626	50,270,280	94.48	98.69	49	48,078,745	35,549,614	12,529,131
Tn_M10_1	51,597,508	48,435,476	93.87	98.65	49	46,185,883	34,049,453	12,136,430
Tn_M10_2	46,148,008	38,698,282	83.86	98.68	49	36,768,620	26,970,121	9,798,499
Tn_M10_3	53,072,922	48,755,468	91.87	98.68	50	46,503,161	34,318,561	12,184,600
Tn_M15_1	55,028,300	50,565,852	91.89	98.66	50	47,925,257	35,642,800	12,282,457
Tn_M15_2	53,716,922	50,735,452	94.45	98.62	49	48,221,268	35,614,791	12,606,477
Tn_M15_3	51,681,194	49,056,980	94.92	98.60	49	46,747,927	35,046,514	11,701,413
Tn_M20_1	43,593,546	39,654,096	90.96	98.71	50	36,404,643	27,467,531	8,937,112
Tn_M20_2	52,063,714	47,143,198	90.55	98.69	50	44,501,336	33,059,695	11,441,641
Tn_M20_3	46,321,730	43,761,072	94.47	98.72	50	39,311,722	29,038,917	10,272,805
Tn_M25_1	38,960,298	35,049,058	89.96	98.61	50	33,049,482	24,797,916	8,251,566
Tn_M25_2	50,889,970	48,362,586	95.03	98.77	49	46,018,962	34,380,829	11,638,133
Tn_M25_3	51,634,566	48,881,308	94.67	98.72	49	45,731,102	33,556,695	12,174,407

*Tn_5* tillering node samples at 5 °C, *Tn_0* tillering node samples at 0 °C, *Tn_M5* tillering node samples at -5 °C, *Tn_M10* tillering node samples at -10 °C, *Tn_M15* tillering node samples at -15 °C, *Tn_M20* tillering node samples at -20 °C, *Tn_M25* tillering node samples at -25 °C

### DEGs obtained under cold stress

**Fig. 2:**
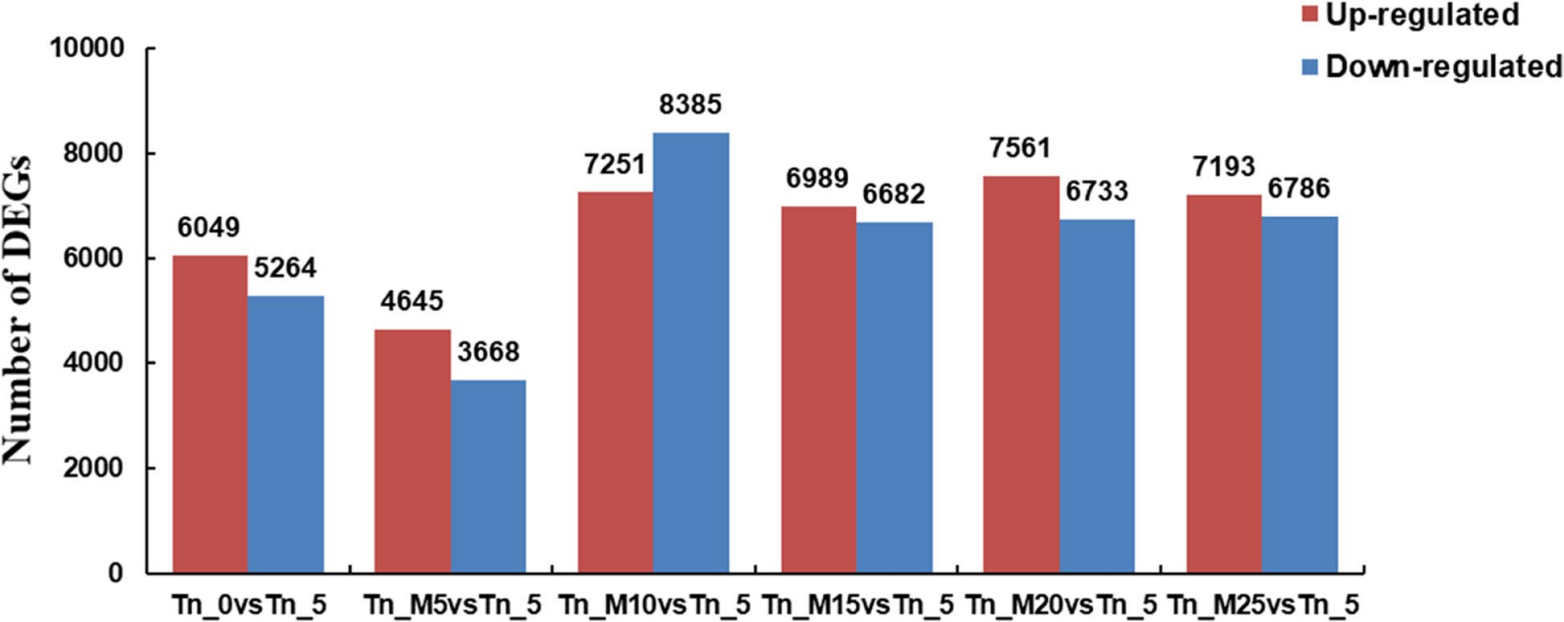
Numbers of DEGs in the tillering node of Dn1 under cold stress. The 5 ℃ condition was included as the control. Tn_5 indicates 5 ℃ samples, Tn_0 indicates 0 ℃ samples, Tn_M5 indicates -5 ℃ samples, Tn_M10 indicates -10 ℃ samples, Tn_M15 indicates -15 ℃ samples, Tn_M20 indicates -20 ℃ samples, Tn_M25 indicates -25 ℃ samples

To understand how the gene expression patterns changed in *Dn1* under cold stress, we performed an analysis of FPKM (fragments per kilobase of exon per million mapped fragments) values across the transcriptome. The specific DEGs at different temperatures were analysed with 5 °C as the control (Table S2). In the 0 ℃ vs. 5 ℃ comparison group, there were 11,313 DEGs (6049 upregulated genes and 5264 downregulated genes). In the -5 ℃ vs. 5 ℃ comparison group, there were 8313 DEGs (4645 upregulated genes and 3668 downregulated genes). In the -10 ℃ vs. 5 ℃ comparison group, there were 15,636 DEGs (7251 upregulated and 8385 downregulated genes). In the -15 ℃ vs. 5 ℃ comparison group, there were 13,671 DEGs (6989 upregulated and 6682 downregulated genes). In the -20 ℃ vs. 5 ℃ comparison group, there were 14,294 DEGs (7561 upregulated and 6733 downregulated genes). In the -25 ℃ vs. 5 ℃ com parison group, there were 14,294 DEGs (7193 upregulated and 6786 downregulated genes). This suggests that these genes are involved in cold resistance (Fig. 2).

**Fig. 3:**
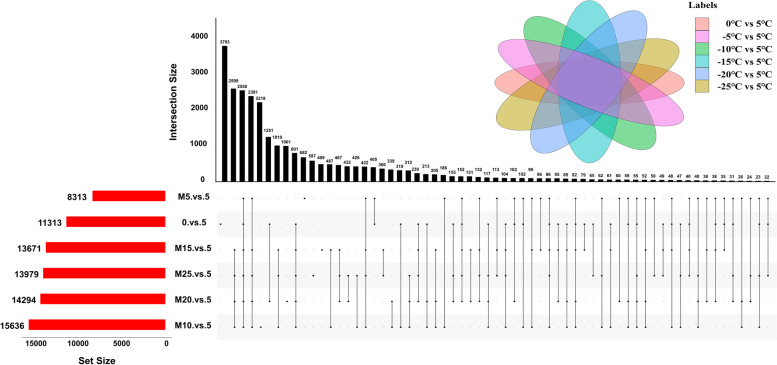
UpSet diagram of DEGs in Dn1 under cold stress. The left bar represents the raw number of each group and the upper bar represents the number of intersections among the groups

In Fig. 3, 2550 common genes were detected in *Dn1* among the six different comparison groups, suggesting that these genes were involved in the process of responding to cold stress at different low temperatures. In addition, a large number of DEGs were generated at extremely low temperatures of -10 ℃, -15 ℃, -20 ℃ and -25 ℃, especially at -10 ℃, compared with other temperatures. A higher number of unique DEGs were generated at 0 ℃, -10 ℃, and -20 ℃.

**Fig. 4:**
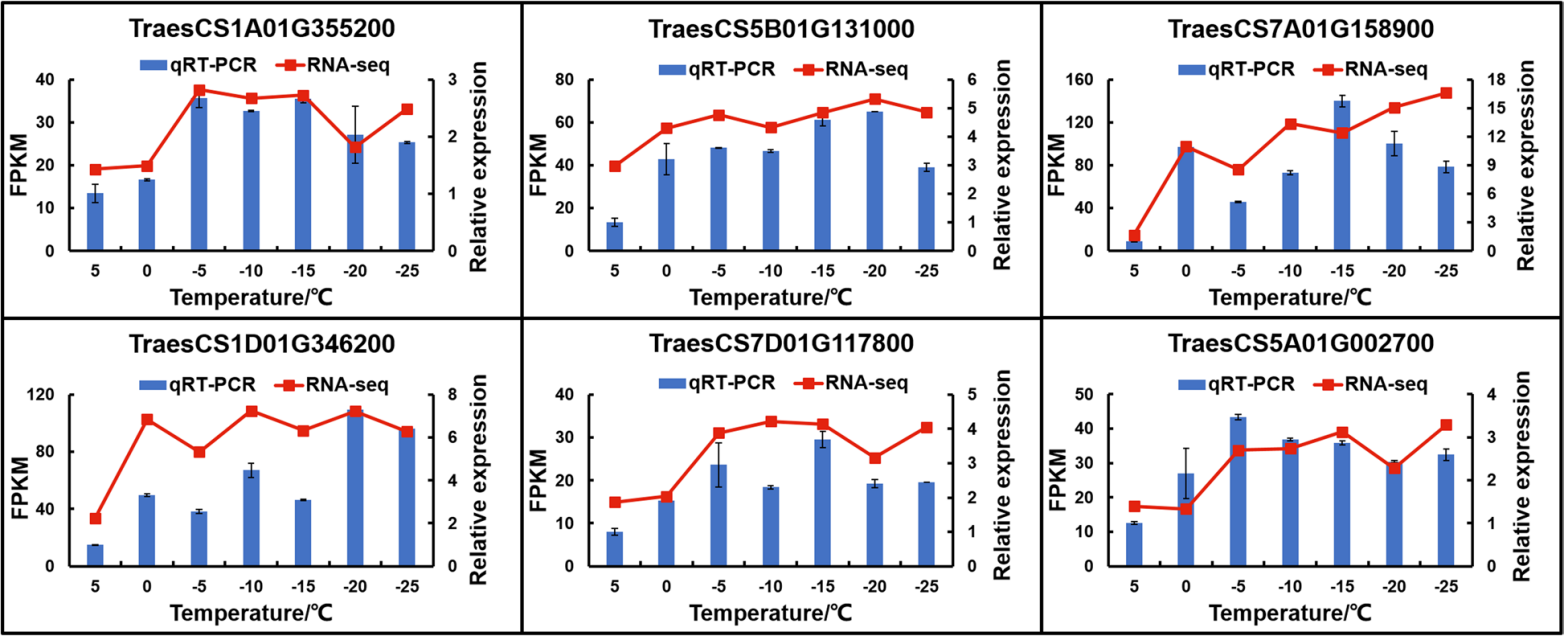
qRT–PCR validation of six DEGs of Dn1

In this study, we randomly selected six DEGs for qRT-PCR validation. The sequences of all the primers needed for the experiments are shown in Table S3. The results showed high congruence (R^2^ = 0.96) between qRT-PCR data and RNA-seq data (Fig. 4 and Fig. S1). This suggests that the RNA-seq data are highly credible for use in further analysis.

### GO and KEGG enrichment analysis of DEGs

**Fig. 5:**
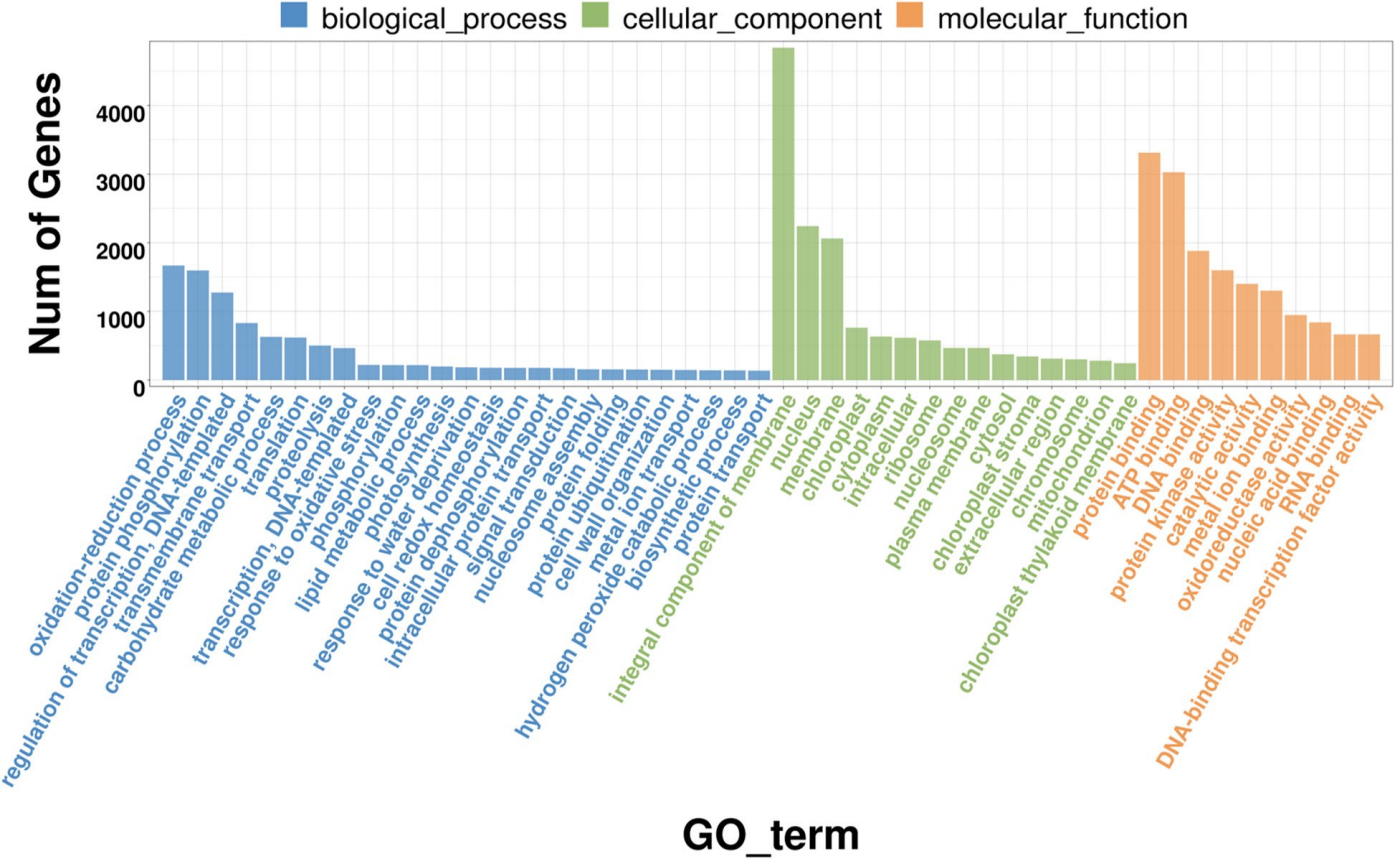
GO analysis of DEGs in the seven temperature comparison groups. The Y and X axes correspond to GO terms and the number of DEGs

*Dn1* produced a large number of DEGs under cold stress. To explore the potential function of DEGs, we performed Gene Ontology (GO) analysis. The results showed that 31,003 DEGs were classified into 3101 GO terms, including three categories: “biological process”, “cellular component” and “molecular function”. The main biological processes were “oxidation–reduction process”, “protein phosphorylation” and “carbohydrate metabolic process”. The main cellular components were “nucleus”, “membrane”, “chloroplast” and “cytoplasm”. The main molecular functions were “binding”, “protein kinase activity” and “catalytic activity” (Fig. 5).

**Fig. 6:**
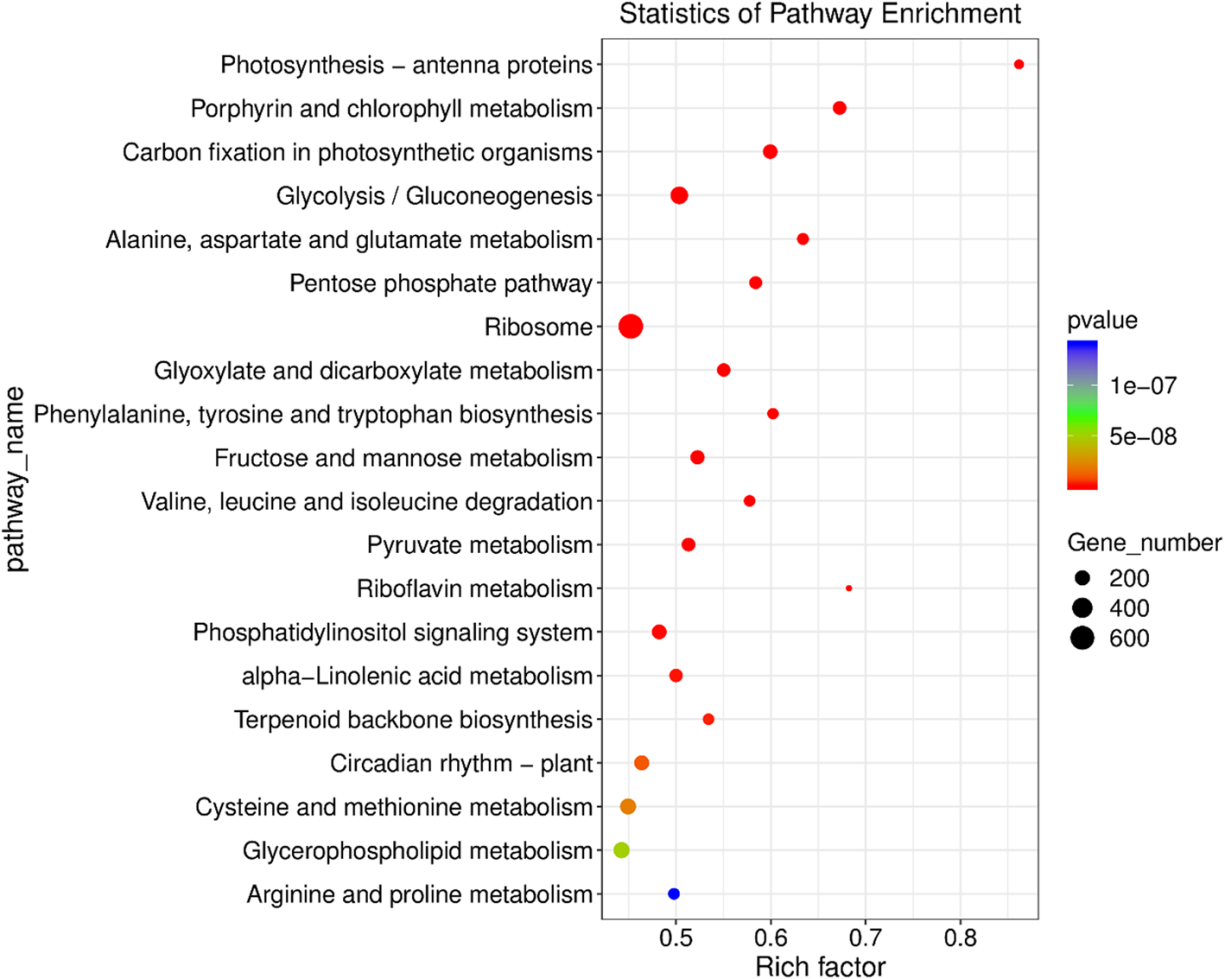
KEGG enrichment analysis of DEGs in the seven temperature comparison groups. The Y axis corresponds to the KEGG pathway, and the X axis shows the enrichment ratio between the number of DEGs enriched in a particular pathway. The colour of the dot represents the p value, and the size of the dot represents the number of DEGs mapped to the reference pathway

To explore the metabolic pathways involved in cold stress in depth, we performed Kyoto Encyclopedia of Genes and Genomes (KEGG) analysis. The 20 common metabolic pathways with the greatest enrichment were exhibited in the comparison of seven temperatures (Fig.6). Among these top pathways, there were many significantly up/downregulated genes involved in carbohydrate metabolism including glycolysis/gluconeogenesis, pentose phosphate pathway and fructose and mannose metabolism. In addition, the MAPK signalling pathway was highly enriched in each of the six comparison groups (0 ℃ vs. 5 ℃, -5 ℃ vs. 5 ℃, -10 ℃ vs. 5 ℃, -15 ℃ vs. 5 ℃, -20 ℃ vs. 5 ℃ and -25 ℃ vs. 5 ℃). Glycolysis/gluconeogenesis was highly enriched at low temperatures, except at -5 and -10 ℃. However, starch and sucrose metabolism was only highly enriched at 0 and -5 ℃. The pentose phosphate pathway and ABC transporters were highly enriched at 0 and -5 ℃, and fructose and mannose metabolism were highly enriched at -10, -15, -20 and -25 ℃. Furthermore, we also found that plant hormone signal transduction was enriched only in the -5 ℃ vs. 5 ℃ comparison group. Briefly, a large number of genes in different pathways are involved in *Dn1* cold resistance. It is possible to deeply explore the *Dn1* cold resistance mechanism from different perspectives (Fig. S2, S3, S4, S5, S6 and S7).

### DEGs involved in cold signal transduction

**Fig. 7:**
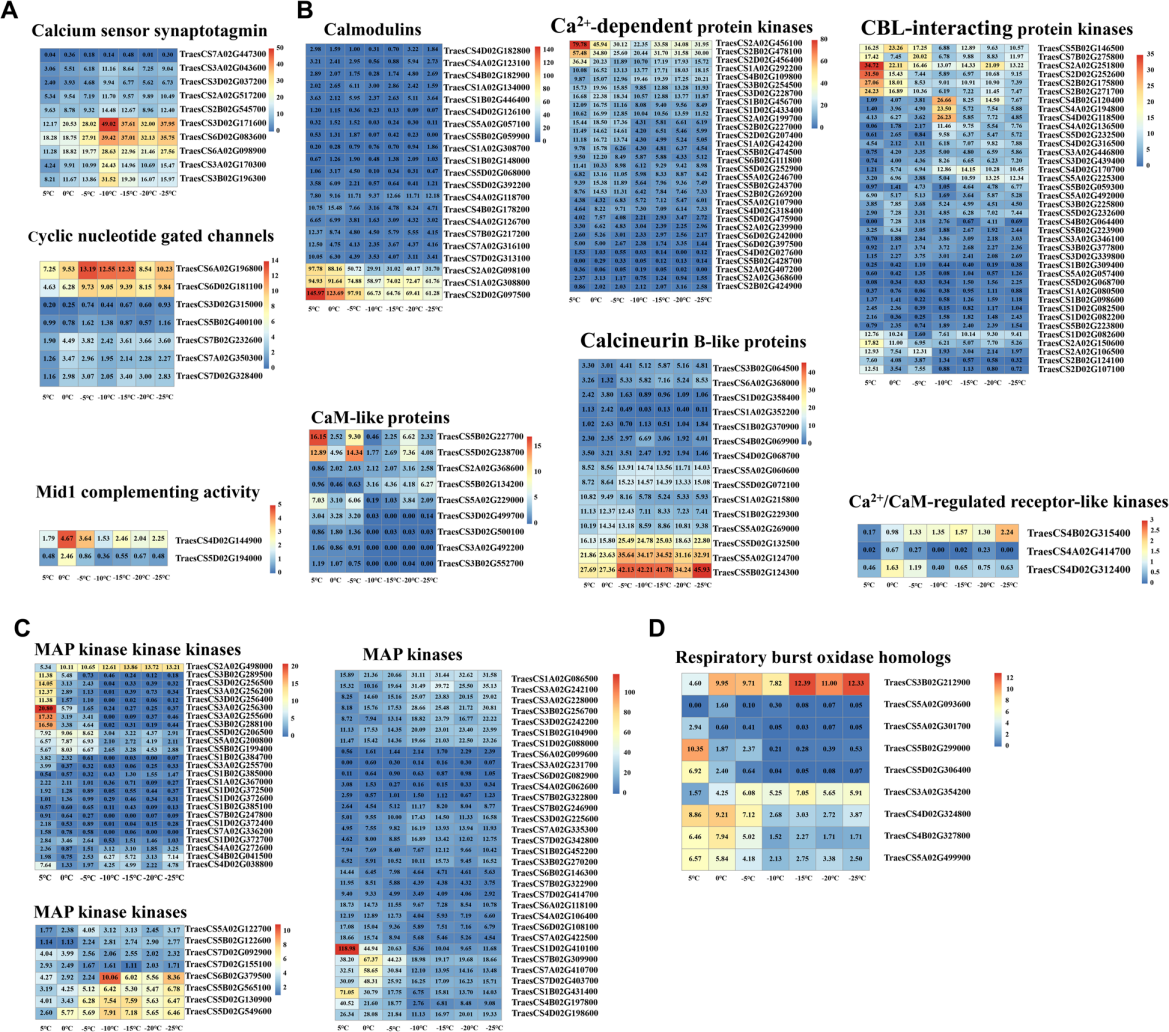
Expression pattern analysis of cold signal transduction-related genes. **A** Expression pattern of genes encoding Ca^2+^ channel proteins. **B** Expression pattern of genes encoding Ca ^2 +^ si gnal transduction-related proteins. **C** Expression pattern of genes encoding MAPK signal cascade-related proteins. **D** Expression pattern of genes encoding RBOH

A rapid increase in Ca^2+^ concentrations in the cytoplasm is considered the earliest cold signalling event. The opening of channels is responsible for the rapid increase in Ca^2+^ concentrations. In *Dn1*, the expression of genes encoding three calcium channels, including mid1 complex activity (*MCAs*), calcium sensor synaptotagmin (*SYTs*) and cyclic nucleotide gated channels (*CNGCs*), was upregulated under cold stress. The calcium signal is transmitted through Ca^2+^ sensors. In *Dn1*, we also found that the expression of genes encoding calcium sensors, including calmodulins (*CaMs*), CaM-like (*CMLs*), Ca^2+^-dependent protein kinases (*CPKs/CDPKs*) and calcineurin B-like (*CBLs*), was upregulated under cold stress. In addition, the expression of genes encoding CBL-interacting protein kinases (*CIPKs*) and Ca^2+^/CaM-regulated receptor-like kinases (*CRLKs*) was also upregulated under cold stress. The mitogen-activated protein kinase (MAPK) signalling cascade positively responds to cold stress. The expression of genes encoding three kinases, including MAP kinase kinase kinases (*MEKKs*), MAP kinase kinases (*MEKs*) and MAP kinases (*MPKs*), was upregulated in *Dn1* under cold stress. Respiratory burst oxidase homologs (RBOHs) are involved in the increase in ROS induction by calcium ions under cold stress. The expression of *RBOHs* was upregulated in *Dn1* under cold stress (Fig. 7).

### Key transcription factors associated with cold stress

**Fig. 8:**
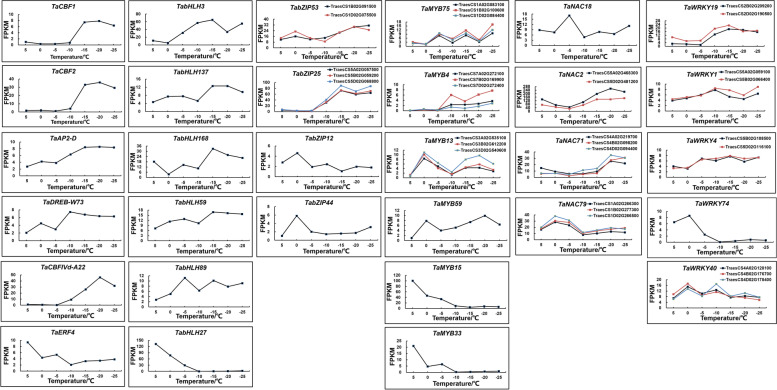
Expression pattern analysis of *AP2/ERF*, *bZIP*, *NAC*, *WRKY*, *bHLH* and *MYB* transcription factor family members

Transcription factors are involved in the regulation of the plant cold response. In this study, we analysed the expression of the *AP2/ERF*, *bZIP*, *NAC*, *WRKY*, *bHLH* and *MYB* transcription factors under cold stress. In all libraries, we found a total of 1021 transcription factors differentially expressed under cold stress (Table S4), which included 85 *bZIP* members (Fig. S8), 93 *NAC* members (Fig. S9), 105 *WRKY* members (Fig. S10), 189 *bHLH* members (Fig. S11), 215 *AP2/ERF* members (Fig. S12) and 334 *MYB* members (Fig. S13). A heatmap was generated to show the FPKM values of differentially expressed transcription factors. Transcription factors with potential research value were selected for illustration. Among the *AP2/ERF* members, the expression of *TaCBF1* (TraesCS5D02G318300), *TaCBF2* (TraesCS5B02G312000), *TaAP2-D* (TraesCS2D02G515800), *TaDREB-W73* (TraesCS3A02G099200), *TaCBFIVd* (TraesCS5A02G310500) and *TaERF5a* (TraesCS5B02G214400) was upregulated at freezing temperatures. The expression of *TaERF4* (TraesCS2D02G543900) and *TaRDFL1a* (TraesCS5D02G200900) was downregulated with decreasing temperature. Among the *bHLH* members, the expression of *TabHLH3* (TraesCS3A02G028200), *TabHLH59* (TraesCS5B02G445900), *TabHLH89* (TraesCS5D02G286300) and *TabHLH137* (TraesCS5A02G292900) was upregulated at freezing temperatures. *TabHLH168* (TraesCS5A02G533300) expression peaked at -15 ℃. The expression of *TabHLH27* (TraesCS2B02G289900) was downregulated with decreasing temperature and barely expressed at temperatures lower than -10 ℃. Among the *bZIP* members, the expression of *TabZIP25* (TraesCS5A02G057500, TraesCS5B02G059200 and TraesCS5D02G068800) and *TabZIP53* (TraesCS1B02G091500 and TraesCS1D02G075500) was upregulated at freezing temperatures. However, *TabZIP12* (TraesCS3B02G411300) and *TabZIP44* (TraesCS5D02G183500) only had increased expression at chilling temperature (0 ℃). Among the *MYB* members, the expression of *TaMYB4* was upregulated at freezing temperatures. The expression of *TaMYB13* (TraesCS3A02G535100, TraesCS3B02G612200 and TraesCS3D02G540600) and *TaMYB59* (TraesCS1B02G055200) was upregulated at low temperatures, especially at 0 ℃ and -20 ℃. *TaMYB75* (TraesCS1A02G083100, TraesCS1B02G100600 and TraesCS1D02G084400) had higher expression at -5 ℃, -15 ℃ and -25 ℃. The expression of *TaMYB15* (TraesCS2D02G378400) and *TaMYB33* (TraesCS6A02G224000) was downregulated with decreasing temperature. Among the *NAC* members, the expression of *TaNAC2* (TraesCS5A02G468300 and TraesCS5D02G481200) and *TaNAC71* (TraesCS4A02G219700, TraesCS4B02G098200 and TraesCS4D02G094400) was upregulated at freezing temperatures. *TaNAC79* (TraesCS1A02G266300, TraesCS1B02G277300 and TraesCS1D02G266500) expression peaked at 0 °C, and *TaNAC18* (TraesCS7A02G263100) expression peaked at -5 °C. Among the *WRKY* members, the expression of *TaWRKY1* (TraesCS5A02G059100 and TraesCS5B02G066400), *TaWRKY4* (TraesCS5B02G108500 and TraesCS5D02G116100) and *TaWRKY19* (TraesCS2B02G209200 and TraesCS2D02G190500) was upregulated at freezing temperatures. *TaWRKY40* (TraesCS4A02G128100, TraesCS4B02G176700 and TraesCS4D02G178400) had higher expression at 0 °C and -10 °C. The expression of *TaWRKY74* (TraesCS5D02G190800) was downregulated with decreasing temperature (Fig. 8).

### DEGs involved in carbohydrate metabolism

**Fig. 9:**
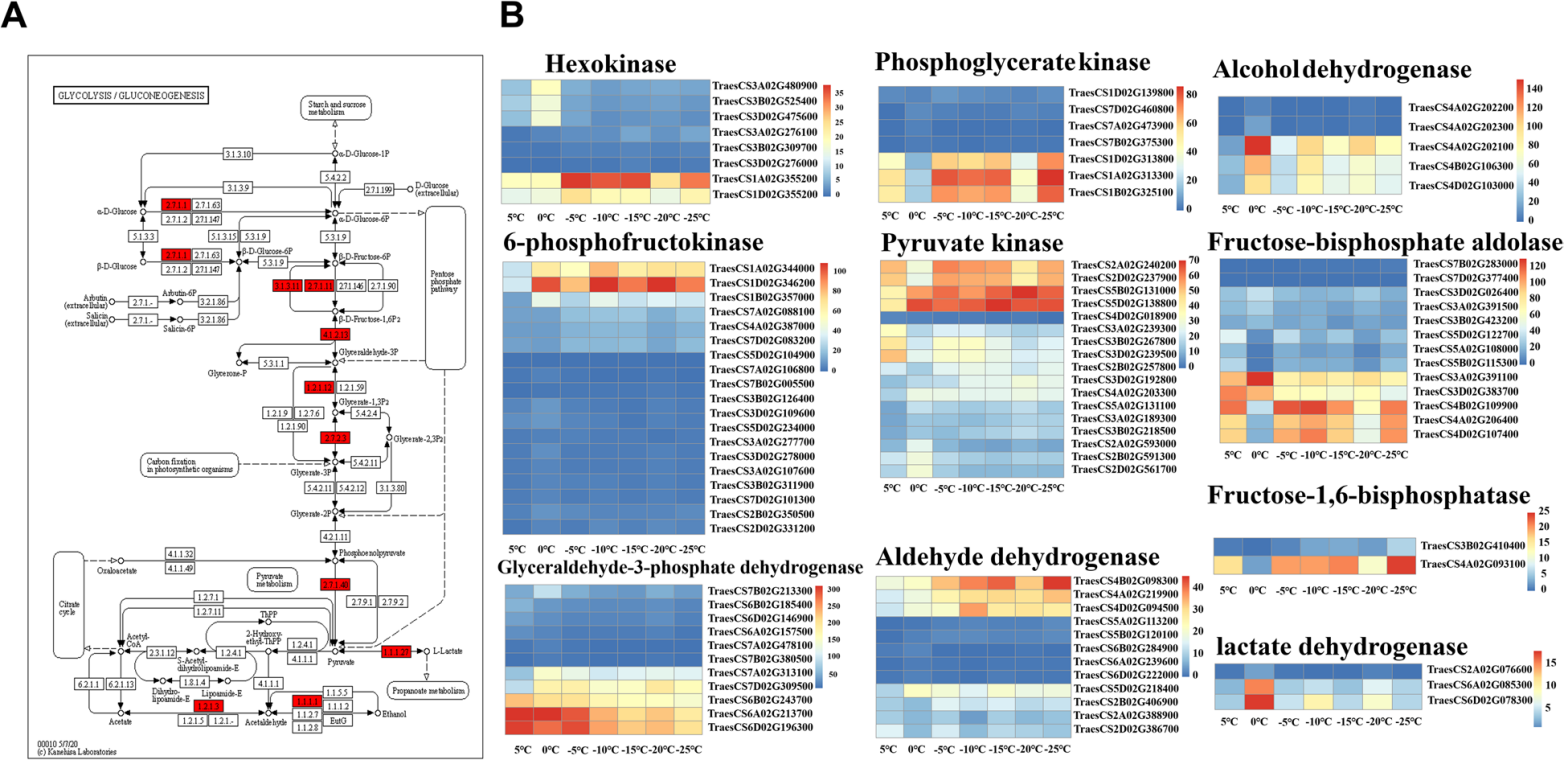
Expression pattern analysis of glycolysis/gluconeogenesis related DEGs under cold stress. (A) KEGG pathway presenting DEG in glycolysis/gluconeogenesis (B) Heatmap presenting DEGs expression patterns

Changes in carbohydrate homeostasis are extremely important for cold resistance when plants are exposed to low temperatures. Through KEGG enrichment analysis, we found a large number of genes involved in carbohydrate metabolism in *Dn1* under cold stress, which included glycolysis/gluconeogenesis, the pentose phosphate pathway, starch and sucrose metabolism and fructose and mannose metabolism. A total of 163 DEGs encoding enzymes related to glycolysis/gluconeogenesis changed significantly under cold stress (Table S5). However, only a few genes encoding related enzymes had higher expression levels under cold stress (Fig. 9). These were hexokinases (EC 2.7.1.1), 6-phosphofructokinases (EC 2.7.1.11), fructose-bisphosphate aldolases (EC 4.1.2.13), glyceraldehyde-3-phosphate dehydrogenases (EC 1.2.1.12), phosphoglycerate kinases (EC 2.7.2.3), pyruvate kinases (EC 2.7.1.40), lactate dehydrogenase (EC 1.1.1.27), alcohol dehydrogenases (EC 1.1.1.1), aldehyde dehydrogenases (EC 1.2.1.3) and fructose-1,6-bisphosphatases (EC 3.1.3.11). Analysis of metabolic pathways revealed that hexokinases, fructose bisphosphate aldolases, and 6-phosphofructokinases were involved in not only glycolysis/gluconeogenesis but also fructose and mannose metabolism. In addition, the gene (TraesCS2B02G599300) encoding phosphomannomutase, which is involved in fructose and mannose metabolism, also had enhanced expression under cold stress. Glucose 6-phosphate dehydrogenase acts as the rate-limiting enzyme of the pentose phosphate pathway, and the gene (TraesCS2A02G320400) encoding this enzyme showed enhanced expression under cold stress.

**Fig. 10:**
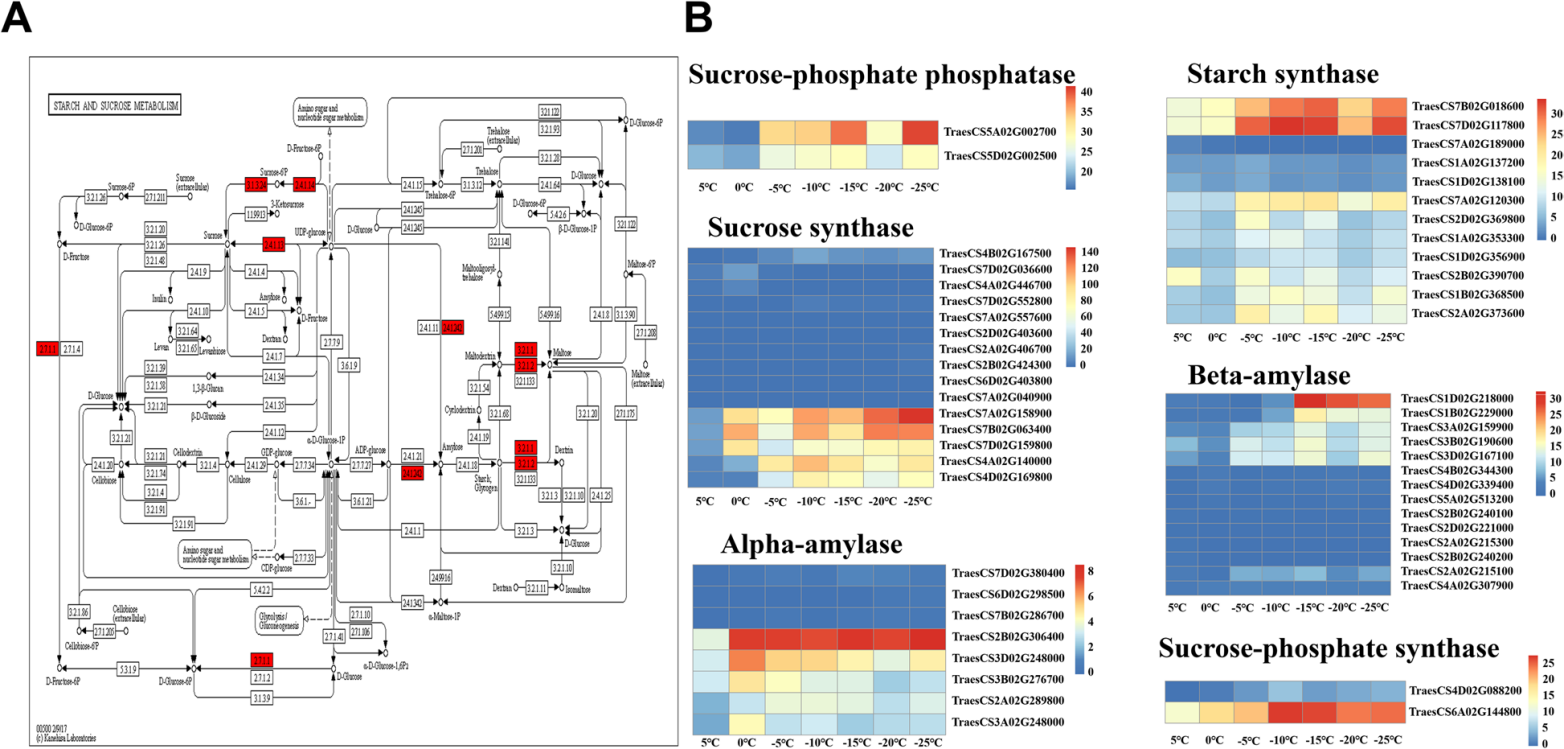
Expression pattern analysis of starch and sucrose metabolism related DEGs under cold stress. (A) KEGG pathway presenting DEGs in starch and sucrose metabolism (B) Heatmap presenting DEG expression patterns

The expression of a total of 62 DEGs encoding enzymes related to starch and sucrose metabolism changed significantly under cold stress (Table S6). In addition to the DEGs encoding hexokinases, only a few genes encoding related metabolic enzymes had higher expression levels under cold stress (Fig. 10). These were sucrose-phosphate synthase (EC 2.4.1.14), sucrose phosphate phosphohydrolase (EC 3.1.3.24), sucrose synthase (EC 2.4.1.13), starch synthases (EC 2.4.1.21), alpha-amylase (EC 3.2.1.1) and beta-amylases (EC 3.2.1.2).

## Discussion

Plants are sessile in nature, so the only way plants can survive in adverse environments is rapid and efficient adaptation to alterations in their surroundings [27]. Cold stress can affect plant growth and development and reduce crop yields [28]. Plant adaptation to cold stress is a complex process that undergoes a series of changes at both the physiological and molecular levels [6]. *Dn1* is a strong cold-resistant winter wheat cultivar able to resist -30 ℃ temperatures in Heilongjiang Province. Understanding how *Dn1* responds to cold stress will provide valuable information and genetic resources for improving cold stress tolerance in crops. Combined with the phenotype of *Dn1* in this study (Fig. 1) and the physiological data determined in our previous study [29], we found that tillering nodes play an important role in the wintering stage of *Dn1*. In this study, we performed transcriptome sequencing of tillering nodes of *Dn1* grown in a field using RNA-seq. The aim was to explore the cold signal perception, cold signal transduction and metabolic process of *Dn1* in response to cold stress at the transcriptional level.

### Signal transduction related genes involved in the *Dn1* response to cold stress

Ca^2+^ is an important second messenger in plants in response to cold stress [30]. The cold stress-triggered Ca^2+^ signal is transmitted mainly by Ca^2+^ sensors, including CaMs, CMLs, CPKs/CDPKs and CBLs [31–33]. It has been reported that the expression of *CaM*s, *CMLs*, *CDPKs* and *CBLs* is upregulated in rice and maize transcriptome under cold stress [34, 35]. In this study, the expression of these Ca^2+^ binding protein genes was also differentially expressed in the *Dn1* transcriptome under cold stress. Studies have revealed that *OsCPK27*, *OsCPK25* and *OsCPK17* are able to induce ROS, nitric oxide and MAPK pathways in response to cold stress [36–39]. In this study, the expression of *CPKs* was mainly significantly upregulated at 0 °C, indicating that these genes may function at chilling temperature. Only one *CPK* gene (TaesCS4B02G109800) was significantly upregulated at freezing temperatures, suggesting that this gene may play a unique role under cold stress. CBLs are able to decode Ca^2+^ signals through interaction with CIPKs, transmitting the signal to downstream phosphorylation events [40, 41]. Sun et al. [40] analysed the physical interaction between TaCBL and TaCIPK proteins in wheat and found that TaCBL2 interacted with TaCIPK11 and TaCIPK14 and that TaCBL6 interacted with TaCIPK11. In this study, we found that the expression of *TaCBL2* (TraesCS5A02G060600 and TraesCS5D02G072100), *TaCBL6* (TraesCS5A02G124700 and TraesCS5B02G124300), *TaCIPK11* (TraesCS3A02G346100, TraesCS3B02G377800 and TraesCS3D02G339800) and *TaCIPK14* (TraesCS4A02G194800, TraesCS4B02G120400 and TraesCS4D02G118500) was upregulated under cold stress. Therefore, we speculate that three CBL-CIPK interactions (TaCBL2-CIPK11, TaCBL2-CIPK14 and TaCBL6-CIPK11) occur under cold stress to transmit Ca^2+^ signals to downstream phosphorylation events. It has been reported that Ca^2+^ regulates CRLK1, a plasma membrane-associated serine/threonine kinase, which plays an important role in the plant response to cold stress [42]. Recent studies have shown that CRLK1 and CRLK2 may regulate the MEKK1-MKK1/2-MPK4 cascade to negatively regulate MPK3/6 in *Arabidopsis thaliana* L. under cold stress [43, 44]. In this study, the expression of *TaCRLK2* (TraesCS4B02G315400), the gene with the highest homology to *AtCRLK2*, was upregulated and the expression of *TaMPK3/6* (TraesCS4A02G106400, TraesCS4B02G197800 and TraesCS4D02G198600) was downregulated under cold stress. However, the expression of *TaMEKK1* (TraesCS5A02G200800 and TraesCS5B02G199400), *TaMKK2* (TraesCS7D02G092900) and *TaMPK4* (TraesCS1A02G184500, TraesCS1B02G192600 and TraesCS7D02G295900) was downregulated or not significantly different under cold stress. These finding suggests that there are differences in regulation mechanisms between wheat and *Arabidopsis*, and TaCRLK2 may not negatively regulate TaMPK3/6 through the TaMEKK1-TaMKK2-TaMPK4 cascade. In addition, we found that the expression of *TaMEK7* (TraesCS2A02G498000), *TaMKK3* (TraesCS5D02G130900 and TraesCS5D02G549600), and *TaMPK16/19/20* (TraesCS1B02G104900, TraesCS1D02G088000, TraesCS3A02G242100, TraesCS7B02G246900, TraesCS3A02G228000, TraesCS3D02G225600 and TraesCS7D02G342800) was upregulated under cold stress (Fig. 7B and C). Among them, the expression of *MKK3* was also upregulated in maize (*Zea mays* L.) and cotton (*Gossypium hirsutum* L.) transcriptomes [45, 46]. This finding suggests that there may be other MAPK signalling cascade that regulates downstream cold-responsive gene expression in *Dn1* under cold stress, which needs further exploration. *RBOH* encodes plasma membrane NADPH oxidase involved in Ca^2+^ induced ROS accumulation under cold stress [47]. In this study, we found that the expression of *RBOHs* (TraesCS3A02G354200 and TraesCS3B02G212900) was upregulated under cold stress, suggesting that an interaction mechanism between Ca^2+^ and ROS signalling may also exist in wheat (Fig. 7D). In summary, the increased expression of Ca^2+^ signalling protein transcripts in *Dn1* activated a complex signalling cascade that regulated various downstream cold responsive genes.

### Transcription factors involved in the *Dn1* response to cold stress

It is well known that functional genes and regulatory genes work synergistically to improve cold tolerance in plants. Among the numerous regulatory genes, TFs play important roles in plants cold response [48]. Over the past two decades, the ICE-CBF-COR signalling pathway has been the best characterized cold stress signalling pathway [8]. Three cold-induced *CBF* genes (*CBF1/DREB1B*, *CBF2/DREB1C* and *CBF3/DREB1A*) in *Arabidopsis* are able to activate the expression of *CORs* to improve plant cold tolerance [49]. Heterologous expression of *CBF1-3* from other species enhances cold tolerance in *Arabidopsis* and tobacco (*Nicotiana tabacum* L.) [50, 51]. In this study, *TaCBF1* and *TaCBF2* had higher expression levels at freezing temperatures (-15 ℃ to -25 ℃). This result indicates that *TaCBF1* and *TaCBF2* not only play a role in cold acclimation, but also improve the freezing tolerance of *Dn1*. Studies have shown that *CBF* genes belonging to CBFIIId, CBFIVa, CBFIVb, CBFIVc and CBFIVd groups display higher constitutive and cold-induced expression in winter cultivars of *Pooideae* compared to spring cultivars [52, 53]. In this study, *TaCBFIIId-A15*, *TaCBFIIId-D19*, *TaCBFIVa-A2*, *TaCBFIVb-D20*, *TaCBFIVb-D21*, *TaCBFIVd-A22*, *TaCBFIVd-D9* and *TaCBFIVd-D22* were differentially expressed in *Dn1* under cold stress (Table S1). The higher constitutive and inducible expression within these CBF groups may play a predominant role in the superior cold tolerance capacity of *Dn1*.

ICE (INDUCER OF CBF EXPRESSION) is a MYC-type bHLH transcription factor. Both ICE1 and ICE2 positively regulate *CBF* expression and freezing tolerance [51, 54]. Studies have shown that *ICE1* is differentially expressed in the transcriptome of cold-tolerant peanut and plays an important role under cold stress [15]. However, in this study, neither *TaICE1* nor *TaICE2* expression changed significantly under cold stress. We hypothesized that TaICE1 and TaICE2 are able to regulate the expression of *TaCBFs* in *Dn1* under cold stress. This finding suggests that the protein activity of TaICE1 and TaICE2 is not entirely determined by the transcriptional level. Studies have shown that ICE1 protein activity is regulated by post-translational modifications in *Arabidopsis* under cold stress. HOS1 and MPK3/6 reduce ICE1 protein activity while SIZ1 and OST1 increase ICE1 protein activity [44, 55–57]. Interestingly, in this study, the expression of *TaHOS1* (TraesCS4A02G247200, TraesCS4B02G067700 and TraesCS4D02G066700) did not significantly change and the expression of *TaMPK3/6* was significantly downregulated, while the expression of *TaSIZ1* (TraesCS1A02G065700, TraesCS1B02G083900 and TraesCS3A02G538000) was significantly upregulated under cold stress. This finding suggests that TaSIZ1 may mediate sumoylation of ICE1/2 to increase its protein activity and subsequently positively regulate *TaCBFs* expression in response to cold stress. Furthermore, the fold change in *TabHLH3* expression is the largest in the bHLH transcription factor family of *Dn1* under cold stress. Therefore, its regulatory mechanism under cold stress warrants further exploration.

MYB15, a member of the R2R3 subfamily of MYB domain proteins, negatively regulates cold-induced *CBFs* [58]. The expression of *TaMYB15*, in contrast to *TaCBF1* and *TaCBF2*, showed a decreasing trend in *Dn1* under cold stress. This suggests that the transcription of *TaMYB15* may be suppressed under cold stress, thereby affecting TaMYB15 activity and attenuating the inhibition of downstream *TaCBFs* expression. In contrast to *TaMYB15*, the expression of *TaMYB4* significantly increased under cold stress. Previous studies have shown that overexpression of *OsMYB4* in *Arabidopsis* enhances cold tolerance of plants [59, 60]. It has also been reported that the bHLH3 and MYB4 interaction ensures the stable accumulation of flavonoids in mulberry (*Morus alba* L.) fruits [61]. Therefore, we speculate that TabHLH3 and TaMYB4 may interact to regulate cold tolerance in *Dn1*. However, the specific mechanism needs further exploration.

### Carbohydrate metabolism in *Dn1* under cold stress

Changes in carbohydrate content are directly related to the physiological activities of plants, such as photosynthesis, respiration and metabolic processes [62]. Studies have shown that many DEGs in the loquat transcriptome under cold stress are enriched in the glycolysis/gluconeogenesis, pyruvate metabolism, starch and sucrose metabolism, pentose and glucose interconversion and fructose and mannose metabolism pathways [63]. This finding is consistent with our results. In this study, carbohydrate metabolism related DEGs were mainly mapped to glycolysis/gluconeogenesis and starch and sucrose metabolism. Glycolysis/gluconeogenesis is an important pathway that fuels respiration, and glucose and fructose further metabolism through this process provides energy (ATP) and reducing power (NADH) to plant cells [64]. Studies have shown that glycolysis is more sensitive to cold stress in cotton, *Cucumis melo* L. and *Eucalyptus nitens* (H. Deane & Maiden) Maiden, and the expression of genes encoding the three rate limiting enzymes (hexokinase, 6-phosphofructokinase and pyruvate kinase) of the glycolysis pathway was significantly upregulated under cold stress [65–67]. Our study showed that the expression of genes encoding hexokinase (TraesCS1A02G355200 and TraesCS1D02G355200), 6-phosphofructokinase (TraesCS1A02G344000 and TraesCS1D02G346200) and pyruvate kinase (TraesCS2A02G240200, TraesCS2D02G237900, TraesCS5B02G131000 and TraesCS5D02G138800) was also significantly upregulated under cold stress (Fig. 9). This finding suggests that these genes may play important roles in the glycolysis of *Dn1* under cold stress and promote the metabolism of glucose and fructose to provide ATP and NADH to plants.

The accumulation of sucrose and soluble sugar in plants under cold stress plays an important role in osmoprotection, maintenance of membrane stability, protein stability, carbohydrate homeostasis and intracellular expansion [68–70]. Jiang et al. [71] found that the expression of eight genes encoding sucrose synthase and two genes encoding beta-amylases was significantly upregulated in the transcriptome of cold-tolerant wheat, the sucrose content and soluble sugar content were also upregulated in cold-tolerant wheat under cold stress. Wang et al. [72] also found that the expression of *SUS6*, *SPS1*, *AMY3* and *BAM2* was significantly upregulated in the transcriptome of cold-tolerant *Brassica campestris* L. and the soluble sugar content was also upregulated under cold stress, meanwhile starch synthesis was not inhibited. In addition, Dong et al. [9] found that cold-tolerant cultivar of *Poa pratensis* under cold stress were able to accumulate more carbohydrates and had greater ROS scavenging ability compared with cold-sensitive cultivar. In our previous study, the sucrose content and soluble sugar content significantly increased in *Dn1* under cold stress, and the ROS scavenging ability was also significantly higher than that of the weak cold-tolerant cultivar [29, 73]. In this study, the expression of genes encoding sucrose phosphate synthase (TraesCS6A02G144800), sucrose phosphate phosphatase (TraesCS5A02G002700 and TraesCS5D02G002500), sucrose synthase (TraesCS7A02G158900, TraesCS7B02G063400, TraesCS7D02G159800, TraesCS4A02G140000 and TraesCS4D02G169800), starch synthase (TraesCS7B02G018600 and TraesCS7D02G117800), alpha-amylase (TraesCS2B02G306400 and TraesCS3D02G248000) and beta-amylase (TraesCS1D02G218000) was upregulated under cold stress (Fig. 10). Changes in gene expression of starch and sucrose metabolism key enzymes in *Dn1* may contribute to the accumulation of carbohydrates, thereby improve ROS scavenging ability under cold stress. In addition, the accumulation of soluble sugars may be at least partially triggered by starch degradation under cold stress [74]. In our previous study, the starch content in *Dn1* decreased at -9 °C and subsequently increased at -18 °C [75]. This suggests that *Dn1* is able to promptly replenish consumed starch under cold stress. The upregulated expression of starch synthase genes in our study may guarantee that starch synthesis is not inhibited when accumulating soluble sugars under cold stress. However, the specific mechanism needs further exploration.

We found many differentially expressed cold responsive genes through the *Dn1* transcriptome. Among them, the expression patterns of carbohydrate metabolism key enzyme genes were consistent with the *Dn1* physiological indexes changes in previous studies. However, their cold tolerance regulatory mechanism remains unclear. Further studies should explore the specific function of these key enzyme genes under cold stress. In addition, the regulatory mechanisms of some transcription factors, such as TaCBFIVd-A22, TabHLH3 and TaMYB4 also need further exploration, enriching the regulatory network of *Dn1* under cold stress.

## Conclusion

Our study is the first comprehensive analysis of the cold resistance mechanism of *Dn1* at the transcriptional level via RNA-seq technology. The important genes involved in cold signal transduction were differentially expressed in *Dn1* under cold stress. A large number of differentially expressed transcription factors belonging to the AP2/ERF, bZIP, NAC, WRKY, bHLH and MYB families were found under cold stress. However, only a few transcription factors were significantly up/downregulated under cold stress. Furthermore, some genes encoding key enzymes of carbohydrate metabolism were upregulated under cold stress, suggesting that carbohydrate metabolism may be beneficial for improving plant cold tolerance. These results provide directions for further exploring the cold resistance mechanism of *Dn1* and provide new ideas for breeding cold resistant crops.

## Materials and methods

### Plant materials and cold treatment

The tested winter wheat variety *Dn1* was obtained from the Northeast Agriculture University Wheat Breeding Laboratory. *Dn1* seeds were sown in a Northeast Agricultural University experimental field (45°7′ N, 126°6′ E) in Heilongjiang Province on September 10, 2019. The tillering nodes were then collected at 5 ℃ (October 1, 2019), 0 ℃ (November 1, 2019), -5 ℃ (November 10, 2019), -10 ℃ (November 18, 2019), -15 ℃ (November 28, 2019), -20 ℃ (December 24, 2019) and -25 ℃ (December 31, 2019) (average minimum temperature for ten consecutive days) and subsequently stored at -80 ℃. The field meteorological data during planting to the completion of sampling have been presented in Fig. S14.

### mRNA library construction and sequencing

Total RNA was extracted using TRIzol reagent (Invitrogen, CA, USA) following the manufacturer's procedure. The total RNA quantity and purity were analysed with a Bioanalyzer 2100 and RNA 6000 Nano LabChip Kit (Agilent, CA, USA) with RNA integrity number > 7.0. Then, the cleaved RNA fragments were reverse-transcribed to create the final cDNA library in accordance with the protocol for the mRNA-Seq sample preparation kit (Illumina, San Diego, USA), and the average insert size for the paired-end libraries was 300 bp (± 50 bp).

### RNA sequencing and read mapping

We performed paired-end sequencing on an Illumina HiSeq 4000 (LC Sciences, USA) following the vendor’s recommended protocol. We aligned reads of all samples to the wheat reference genome (ftp://ftp.ensemblgenomes.org/pub/release-45/plants/fasta/triticum_aestivum/dna) using the HISAT package. HISAT allows multiple alignments per read (up to 20 by default) and a maximum of two mismatches when mapping the reads to the reference sequence. HISAT builds a database of potential splice junctions and confirms these by comparing the previously unmapped reads to the database of putative junctions.

### Transcript abundance estimation and differential expression analysis

The mapped reads of each sample were assembled using StringTie. Then, all transcriptomes from samples were merged to reconstruct a comprehensive transcriptome using Perl scripts. After the final transcriptome was generated, StringTie and edgeR were used to estimate the expression levels of all transcripts. StringTie was used to determine the expression level of mRNAs by calculating FPKM. The differentially expressed mRNAs and genes were selected according to log_2_ (fold change) > 1 or log_2_ (fold change) < -1 and statistical significance (*p* value < 0.05) by edgeR.

### GO and KEGG enrichment analysis

GO term enrichment of DEGs was analysed with the GO database (http://www.geneo ntology.org). The significantly enriched metabolic pathways of the DEGs were analysed with the KEGG database (http://www.kegg.jp/kegg).

### Quantitative real-time PCR

Total RNA was extracted from the plants using an Ultrapure RNA Kit (CWBIO, Jiangsu, China). cDNA was subsequently obtained from mRNA with a HiScript III 1st Strand cDNA Synthesis Kit (Vazyme, Nanjing, China). Quantitative real-time PCR (qRT-PCR) was performed using ChamQ Universal SYBR qPCR Master Mix (Vazyme) according to the manufacturer’s instructions. *TaACTIN* was selected as an internal reference gene for wheat. The experimental results were analysed by the 2^−ΔΔCT^ method. qRT-PCR analysis included three independent technical repeats with three biological replicates. The sequences of all the primers needed for the experiments are shown in Table S7.

## Supplementary Information

**Additional file 1:** **Fig. S1.** Correlation analysis between qRT-PCR and RNA-Seq data based on log_2_(fold change) of sixselected genes. **Fig. S2.** KEGG enrichment analysis of DEGs in the Tn_0 vs. Tn_5 comparison group. The Y axis corresponds to KEGG pathway, the X axis shows the enrichment ratio between the number of DEGs enriched in a particular pathway. The color of the dot represents pvalue, and the size of the dot represents the number of DEGs mapped to the referent pathway. **Fig. S3.** KEGG enrichment analysis of DEGs in the Tn_M5 vs. Tn_5 comparison group. The Y axis corresponds to KEGG pathway, the X axis shows the enrichment ratio between the number of DEGs enriched in a particular pathway. The color of the dot represents pvalue, and the size of the dot represents the number of DEGs mapped to the referent pathway. **Fig. S4.** KEGG enrichment analysis of DEGs in the Tn_M10 vs. Tn_5 comparison group. The Y axis corresponds to KEGG pathway, the X axis shows the enrichment ratio between the number of DEGs enriched in a particular pathway. The color of the dot represents pvalue, and the size of the dot represents the number of DEGs mapped to the referent pathway. **Fig. S5.** KEGG enrichment analysis of DEGs in the Tn_M15 vs. Tn_5 comparison group. The Y axis corresponds to KEGG pathway, the X axis shows the enrichment ratio between the number of DEGs enriched in a particular pathway. The color of the dot represents pvalue, and the size of the dot represents the number of DEGs mapped to the referent pathway. **Fig. S6.** KEGG enrichment analysis of DEGs in the Tn_M20 vs. Tn_5 comparison group. The Y axis corresponds to KEGG pathway, the X axis shows the enrichment ratio between the number of DEGs enriched in a particular pathway. The color of the dot represents pvalue, and the size of the dot represents the number of DEGs mapped to the referent pathway. **Fig. S7.** KEGG enrichment analysis of DEGs in the Tn_M25 vs. Tn_5 comparison group. The Y axis corresponds to KEGG pathway, the X axis shows the enrichment ratio between the number of DEGs enriched in a particular pathway. The color of the dot represents pvalue, and the size of the dot represents the number of DEGs mapped to the referent pathway. **Fig. S8.** Expression pattern analysis of differentially expressed *bZIP* transcription factors. **Fig. S9.** Expression pattern analysis of differentially expressed *NAC* transcription factors. **Fig. S10.** Expression pattern analysis of differentially expressed *WRKY* transcription factors. **Fig.S11. **Expression pattern analysis of differentially expressed *bHLH* transcription factors. **Fig. S12.** Expression pattern analysis of differentially expressed *AP2/ERF* transcription factors. **Fig. S13.** Expression pattern analysis of differentially expressed *MYB *transcription factors. **Fig. S14.** The field meteorological data during planting to completion of sampling. (A) Daily minimum and maximum temperatures (B) Daily precipitation.

**Additional file 2.** 

**Additional file 3.** 

**Additional file 4.** 

**Additional file 5.** 

**Additional file 6.** 

**Additional file 7.** 

## Data Availability

Raw Illumina sequence data were deposited in the National Center for Biotechnology Information (NCBI). Upon publication, open access is available in the sequence read archive (SRA) database (https://www.ncbi.nlm.nih.gov/sra). The accession number is PRJNA787922 (https://www.ncbi.nlm.nih.gov/bioproject/PRJNA787922), which includes 21 accession items (SRR17227743-SRR17227763). All data generated or analysed during this study are included in this published article and its supplementary information files.
