# Supplementary material for: Transcriptome analysis of the winter wheat Dn1 in response to cold stress
Source: BMC Plant Biol. 2022 Jun 6;22:277. doi: 10.1186/s12870-022-03654-1 (PMC9169401; doi:10.1186/s12870-022-03654-1)
Supplement: Supplementary file 1 — Additional file 1: Fig. S1. Correlation analysis between qRT-PCR and RNA-Seq data based on log2(fold change) of sixselected genes. Fig. S2. KEGG enrichment analysis of DEGs in the Tn_0 vs. Tn_5 comparison group. The Y axis corresponds to KEGG pathway, the X axis shows the enrichment ratio between the number of DEGs enriched in a particular pathway. The color of the dot represents pvalue, and the size of the dot represents the number of DEGs mapped to the referent pathway. Fig. S3. KEGG enrichment analysis of DEGs in the Tn_M5 vs. Tn_5 comparison group. The Y axis corresponds to KEGG pathway, the X axis shows the enrichment ratio between the number of DEGs enriched in a particular pathway. The color of the dot represents pvalue, and the size of the dot represents the number of DEGs mapped to the referent pathway. Fig. S4. KEGG enrichment analysis of DEGs in the Tn_M10 vs. Tn_5 comparison group. The Y axis corresponds to KEGG pathway, the X axis shows the enrichment ratio between the number of DEGs enriched in a particular pathway. The color of the dot represents pvalue, and the size of the dot represents the number of DEGs mapped to the referent pathway. Fig. S5. KEGG enrichment analysis of DEGs in the Tn_M15 vs. Tn_5 comparison group. The Y axis corresponds to KEGG pathway, the X axis shows the enrichment ratio between the number of DEGs enriched in a particular pathway. The color of the dot represents pvalue, and the size of the dot represents the number of DEGs mapped to the referent pathway. Fig. S6. KEGG enrichment analysis of DEGs in the Tn_M20 vs. Tn_5 comparison group. The Y axis corresponds to KEGG pathway, the X axis shows the enrichment ratio between the number of DEGs enriched in a particular pathway. The color of the dot represents pvalue, and the size of the dot represents the number of DEGs mapped to the referent pathway. Fig. S7. KEGG enrichment analysis of DEGs in the Tn_M25 vs. Tn_5 comparison group. The Y axis corresponds to KEGG pathway, the X axi [file 12870_2022_3654_MOESM1_ESM.docx]

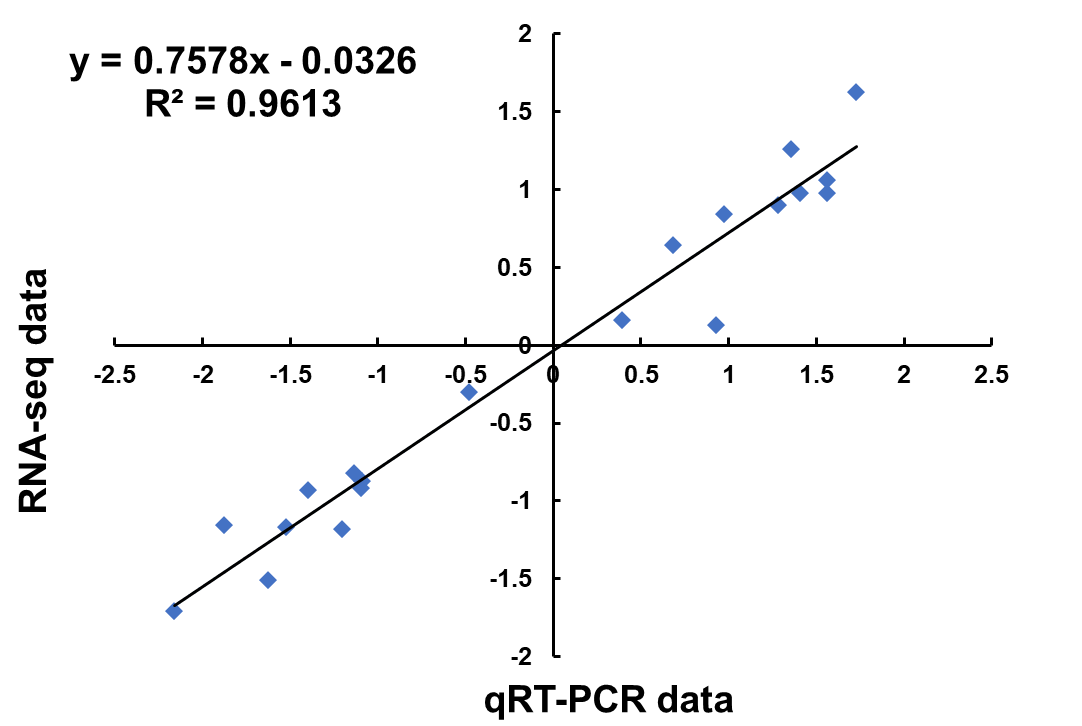


**Fig. S1** Correlation analysis between qRT-PCR and RNA-Seq data based on log_2_ (fold change) of six selected genes.


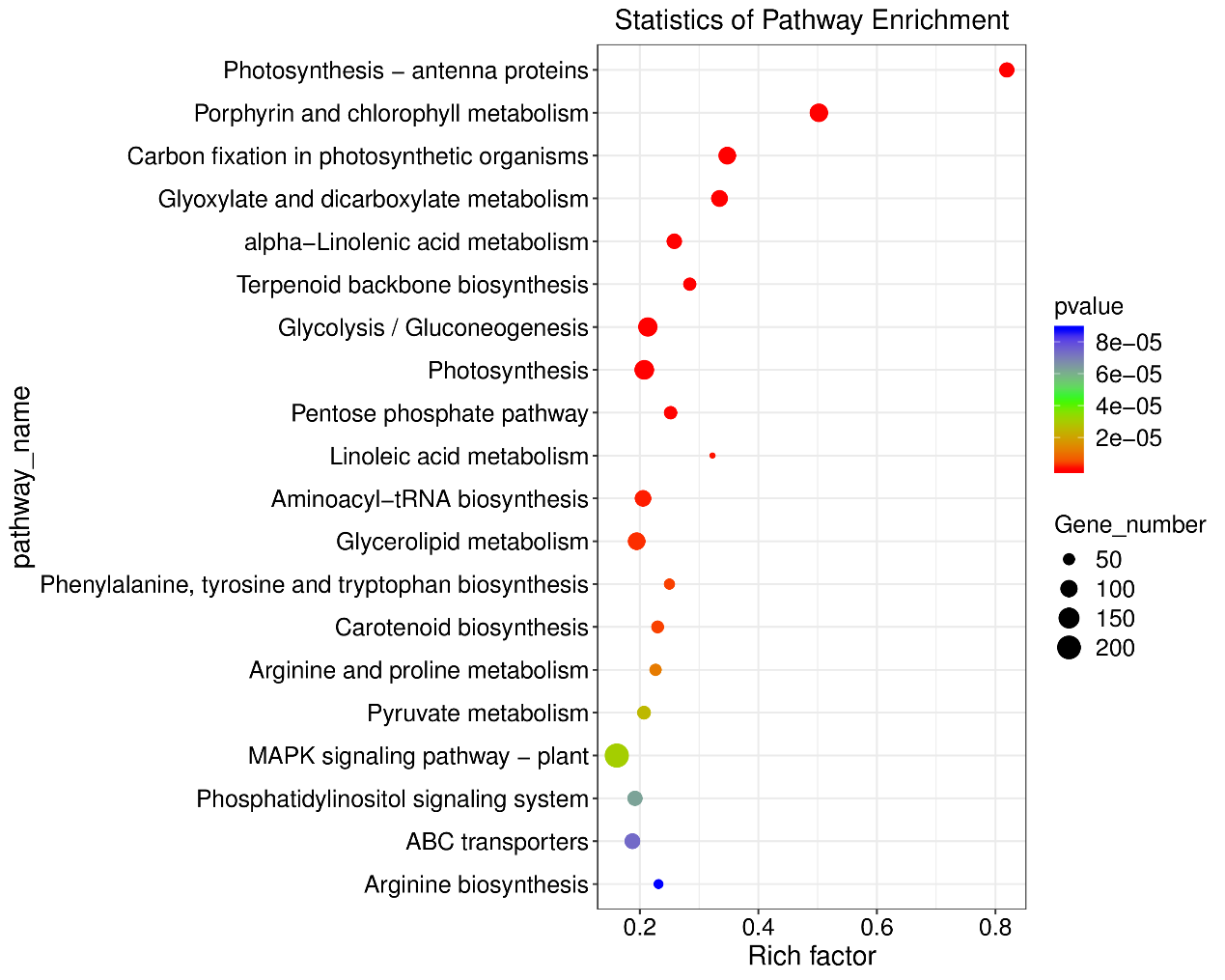


**Fig. S2** KEGG enrichment analysis of DEGs in the Tn_0 vs. Tn_5 comparison group. The Y axis corresponds to KEGG pathway, the X axis shows the enrichment ratio between the number of DEGs enriched in a particular pathway. The color of the dot represents pvalue, and the size of the dot represents the number of DEGs mapped to the referent pathway


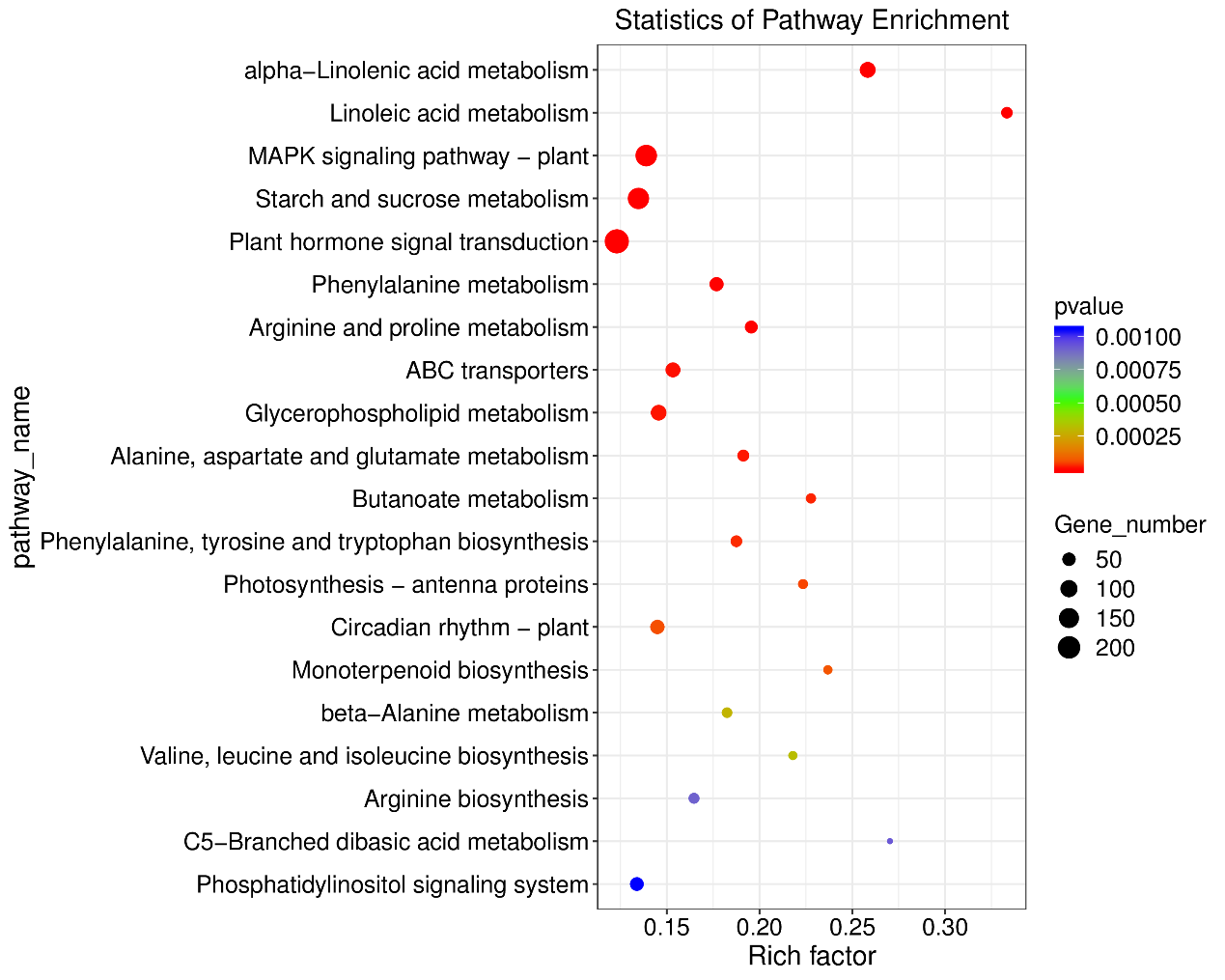


**Fig. S3** KEGG enrichment analysis of DEGs in the Tn_M5 vs. Tn_5 comparison group. The Y axis corresponds to KEGG pathway, the X axis shows the enrichment ratio between the number of DEGs enriched in a particular pathway. The color of the dot represents pvalue, and the size of the dot represents the number of DEGs mapped to the referent pathway


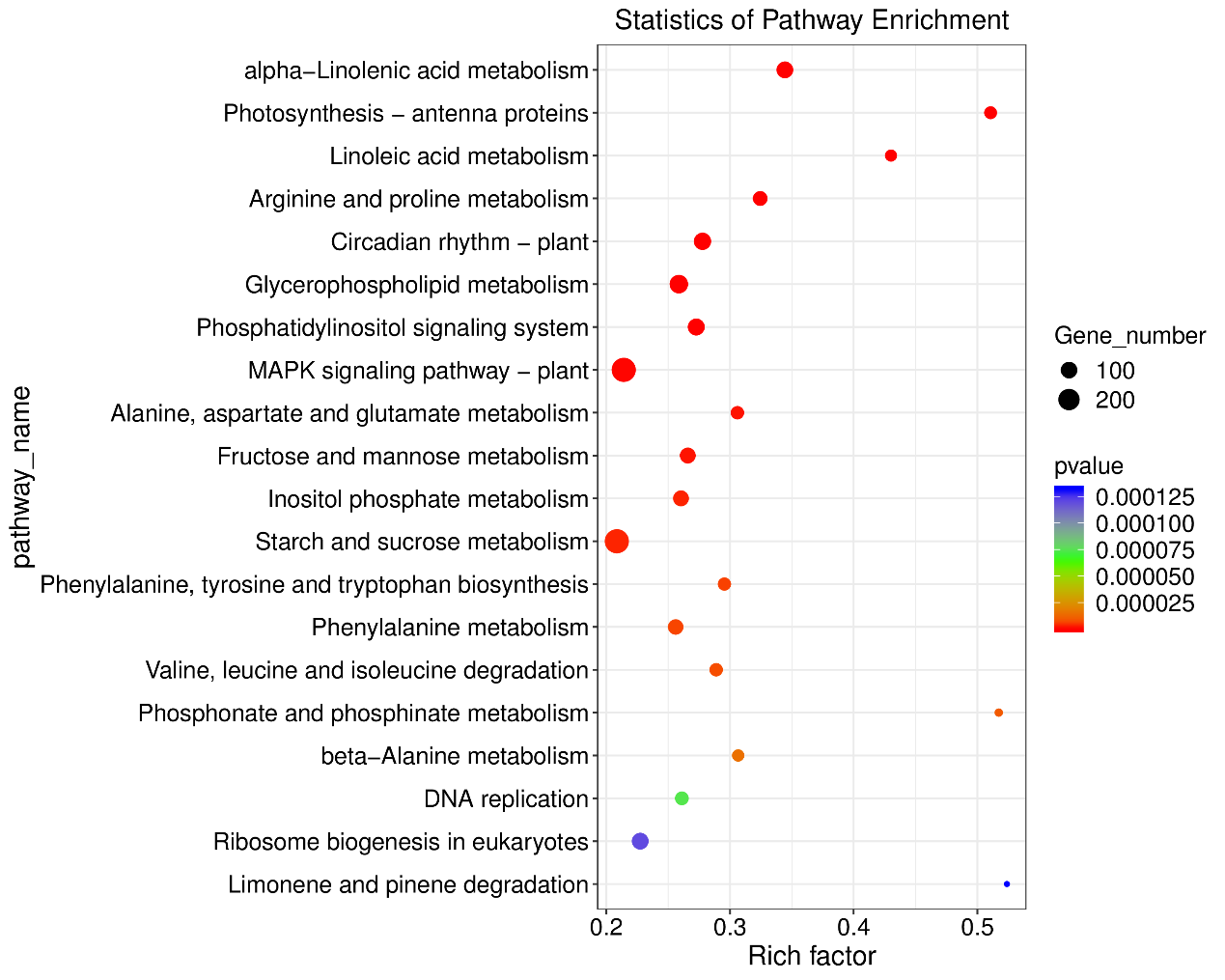


**Fig. S4** KEGG enrichment analysis of DEGs in the Tn_M10 vs. Tn_5 comparison group. The Y axis corresponds to KEGG pathway, the X axis shows the enrichment ratio between the number of DEGs enriched in a particular pathway. The color of the dot represents pvalue, and the size of the dot represents the number of DEGs mapped to the referent pathway


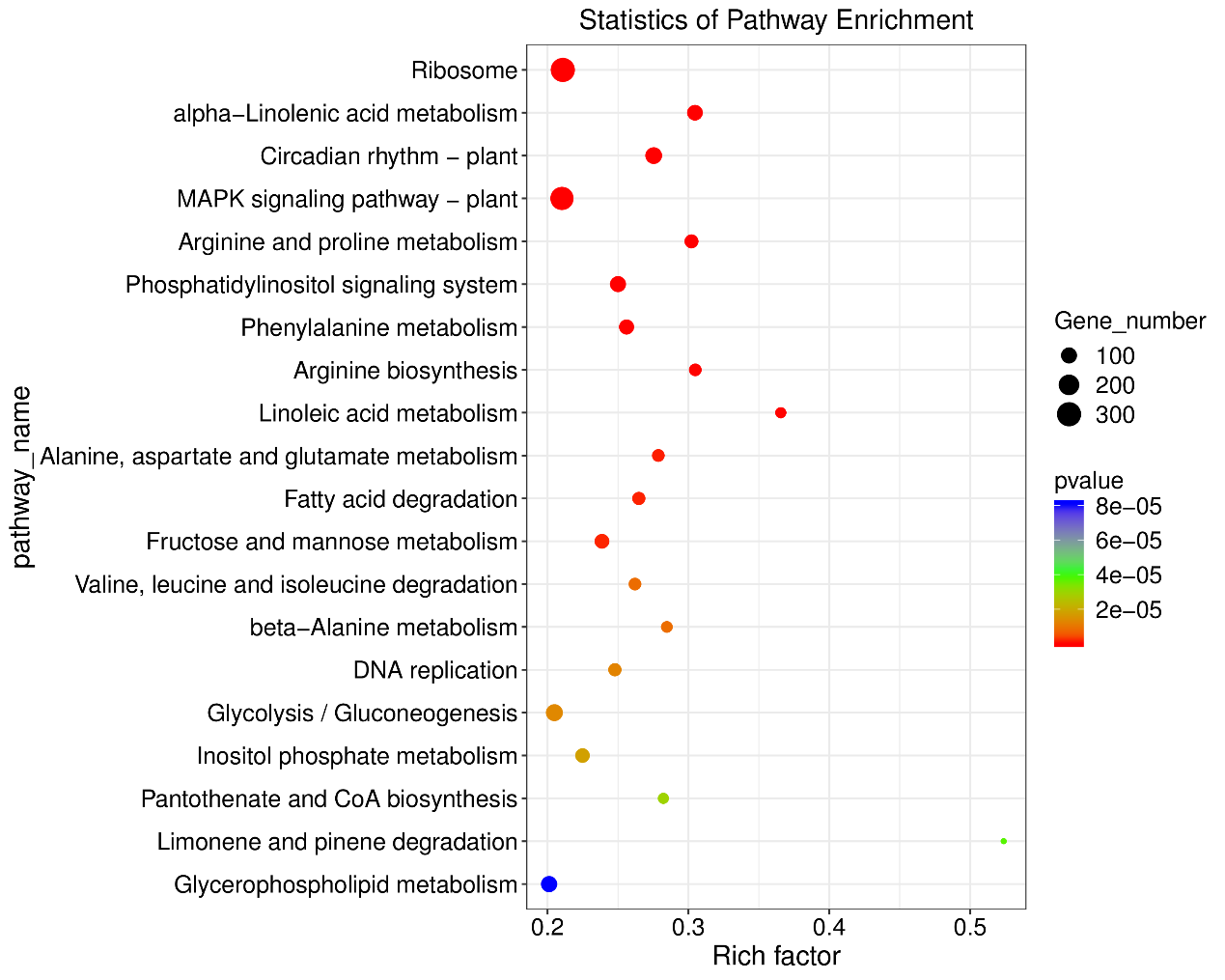


**Fig. S5** KEGG enrichment analysis of DEGs in the Tn_M15 vs. Tn_5 comparison group. The Y axis corresponds to KEGG pathway, the X axis shows the enrichment ratio between the number of DEGs enriched in a particular pathway. The color of the dot represents pvalue, and the size of the dot represents the number of DEGs mapped to the referent pathway


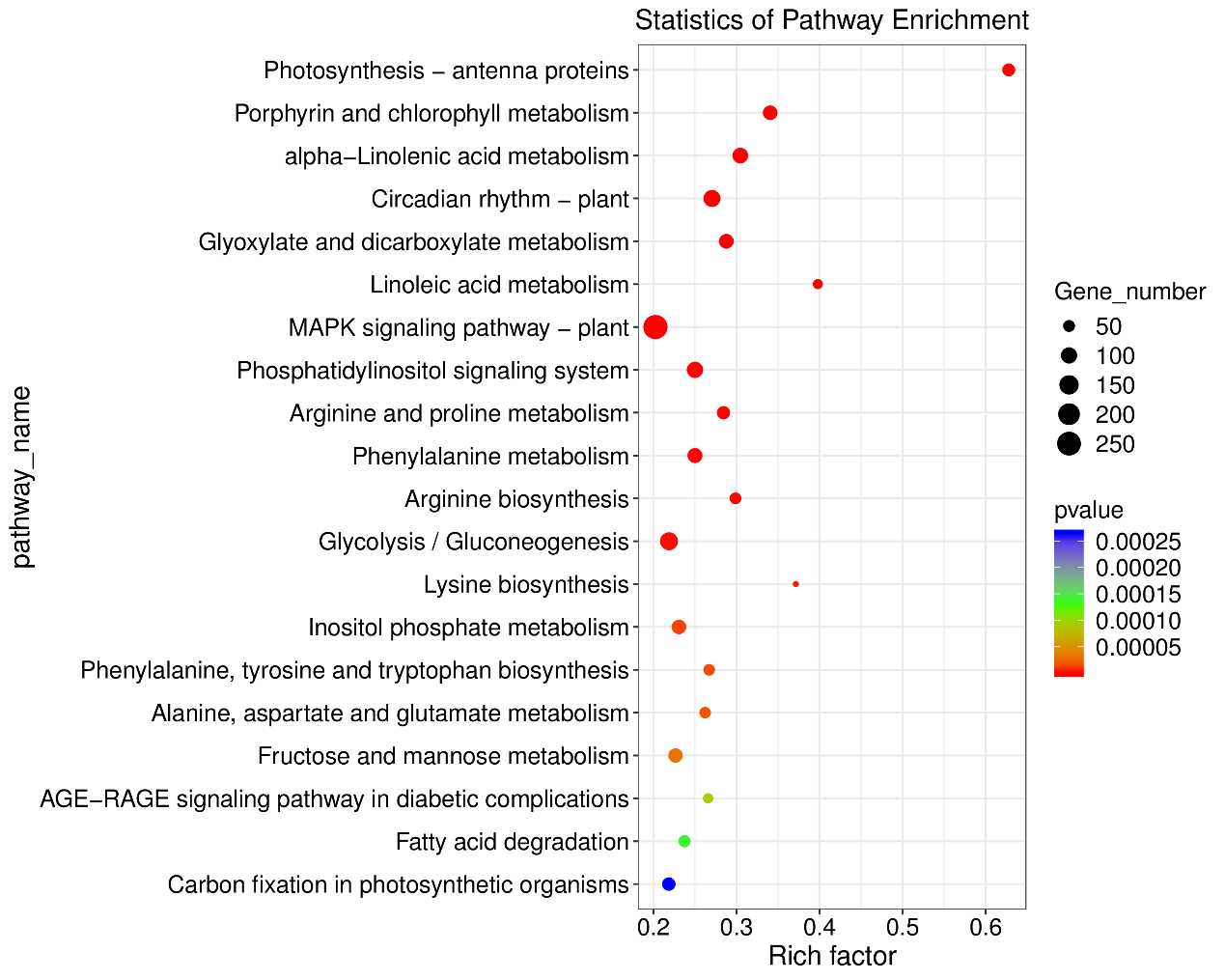


**Fig. S6** KEGG enrichment analysis of DEGs in the Tn_M20 vs. Tn_5 comparison group. The Y axis corresponds to KEGG pathway, the X axis shows the enrichment ratio between the number of DEGs enriched in a particular pathway. The color of the dot represents pvalue, and the size of the dot represents the number of DEGs mapped to the referent pathway


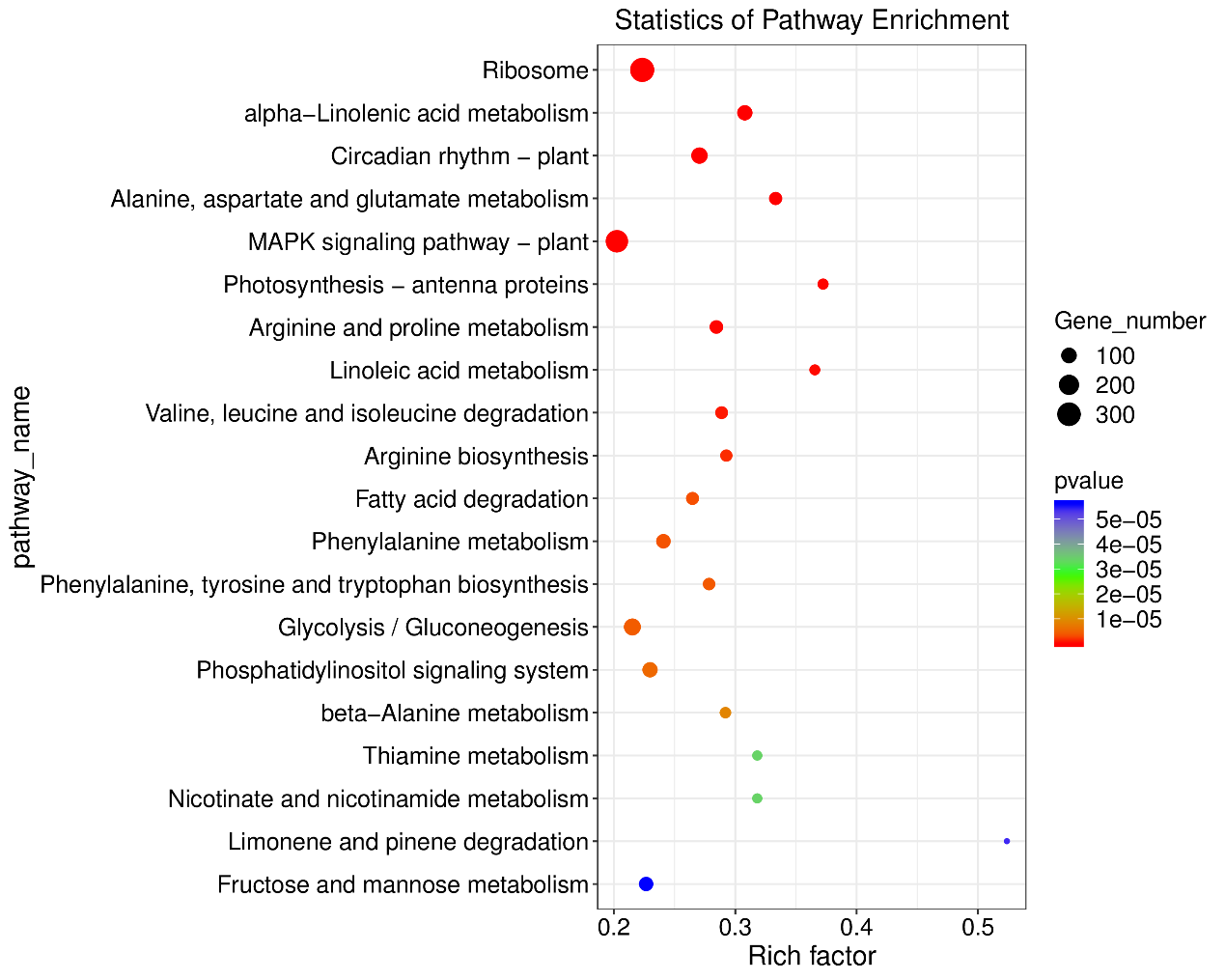


**Fig. S7** KEGG enrichment analysis of DEGs in the Tn_M25 vs. Tn_5 comparison group. The Y axis corresponds to KEGG pathway, the X axis shows the enrichment ratio between the number of DEGs enriched in a particular pathway. The color of the dot represents pvalue, and the size of the dot represents the number of DEGs mapped to the referent pathway


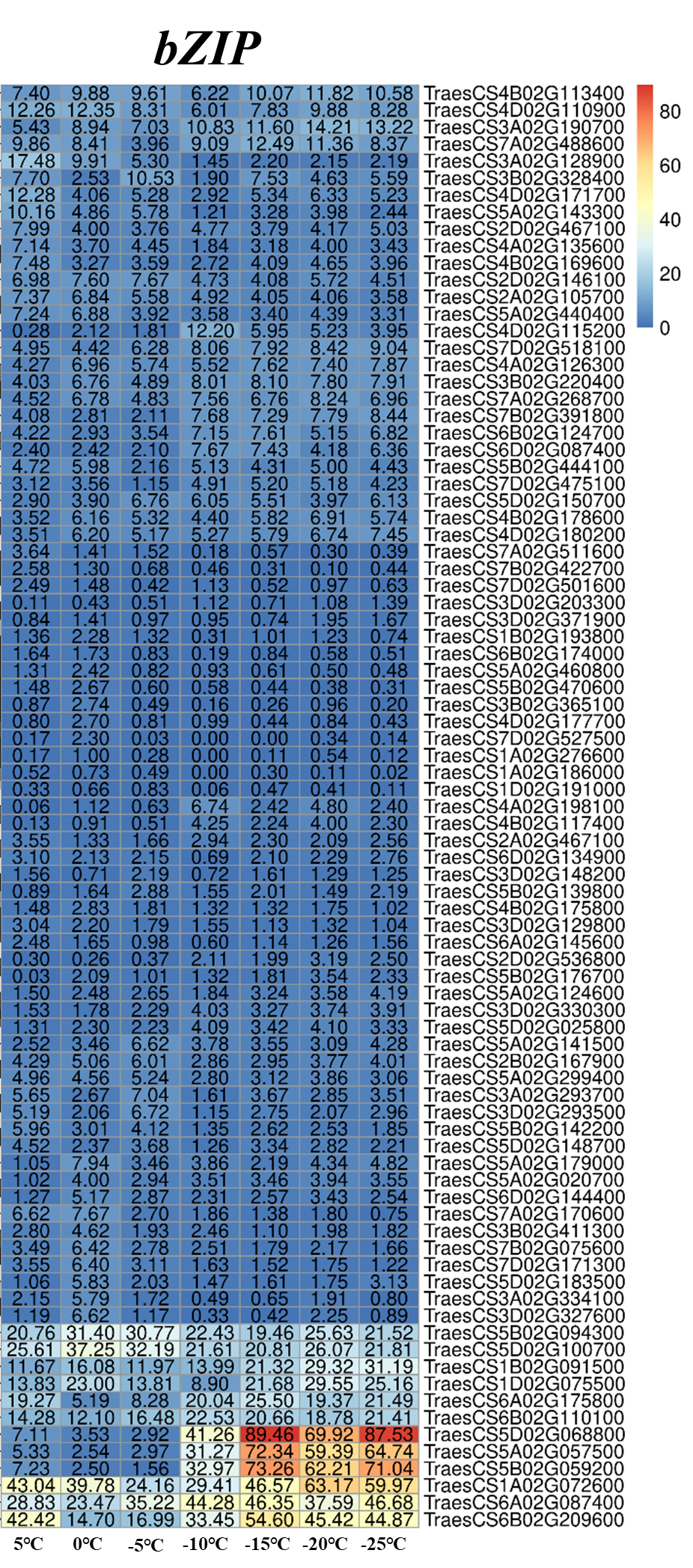


**Fig. S8** Expression pattern analysis of differentially expressed *bZIP* transcription factors.


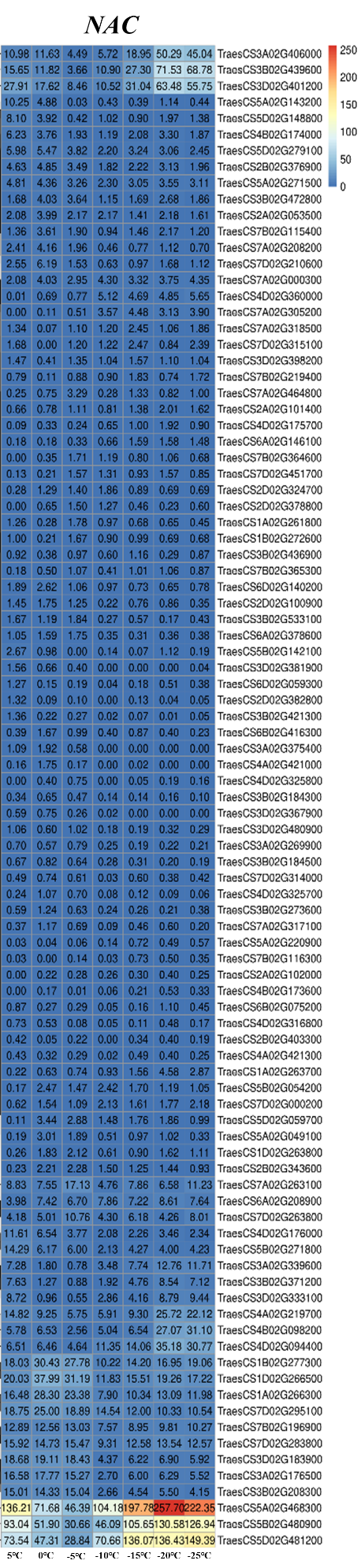


**Fig. S9** Expression pattern analysis of differentially expressed *NAC* transcription factors.


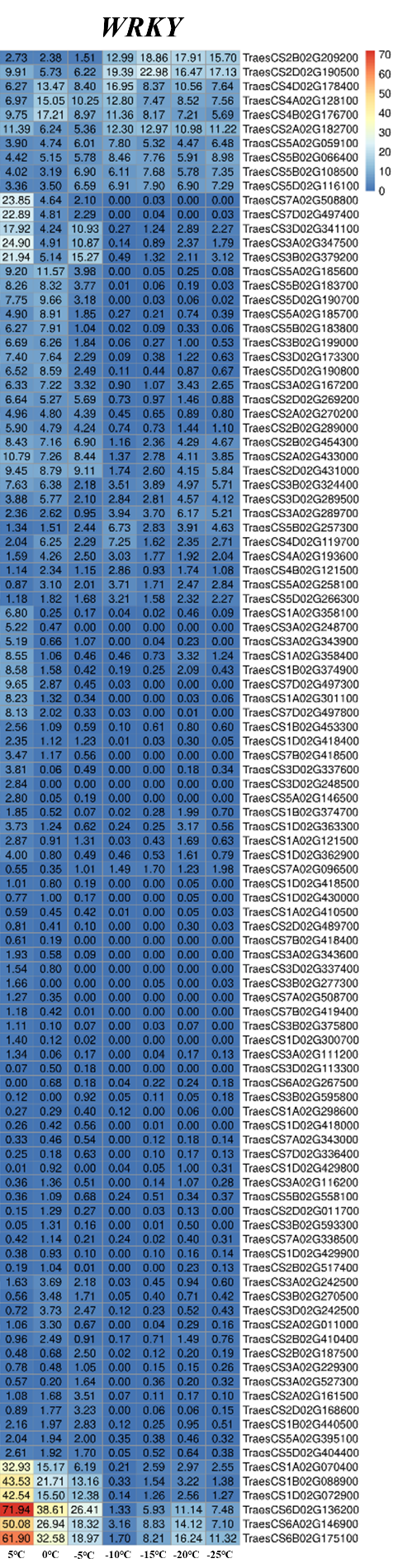


**Fig. S10** Expression pattern analysis of differentially expressed *WRKY* transcription factors.


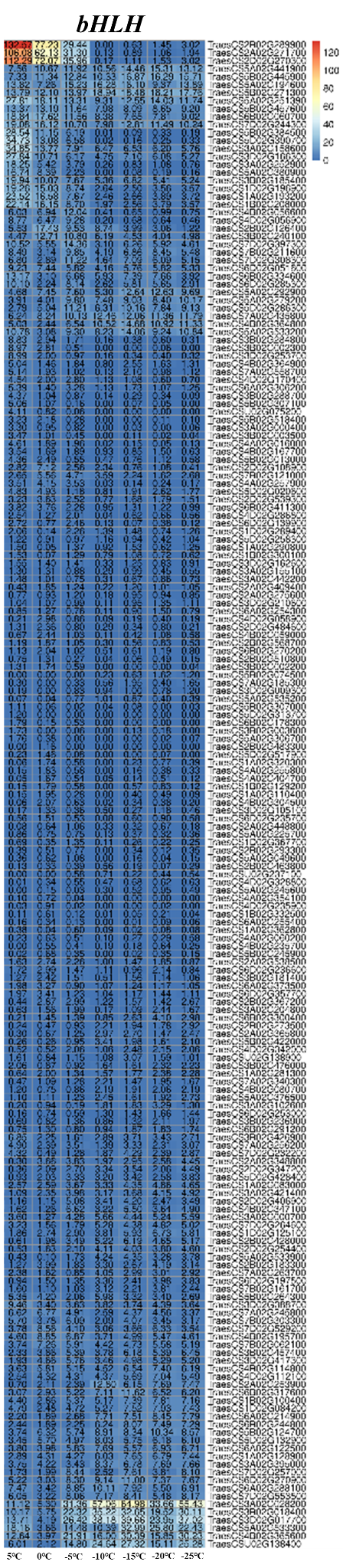


**Fig. S11** Expression pattern analysis of differentially expressed *bHLH* transcription factors.


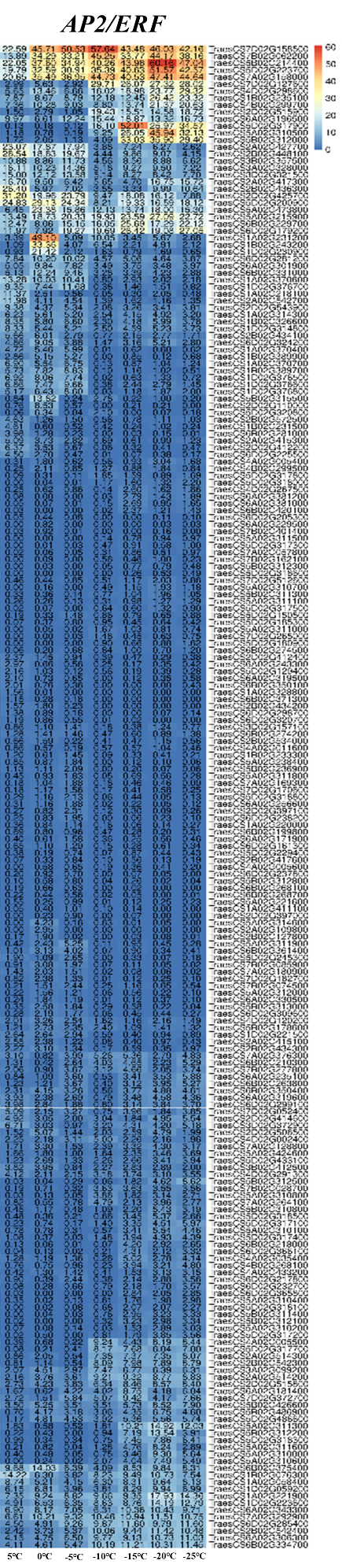


**Fig. S12** Expression pattern analysis of differentially expressed *AP2/ERF* transcription factors


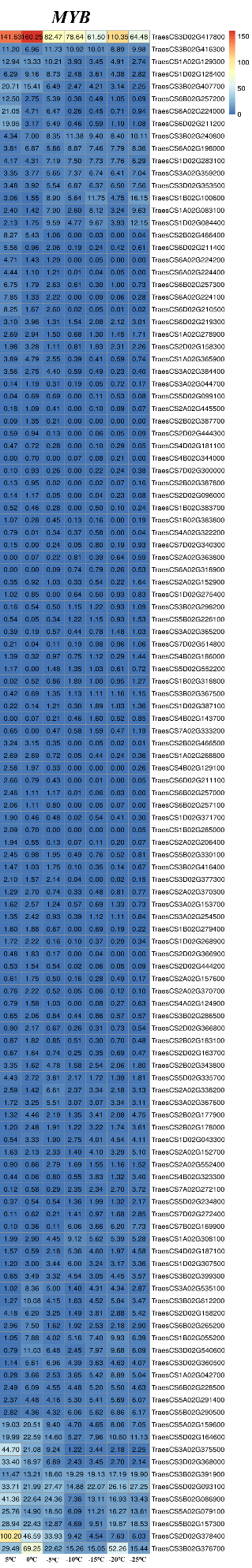


**Fig. S13** Expression pattern analysis of differentially expressed *MYB* transcription factors.


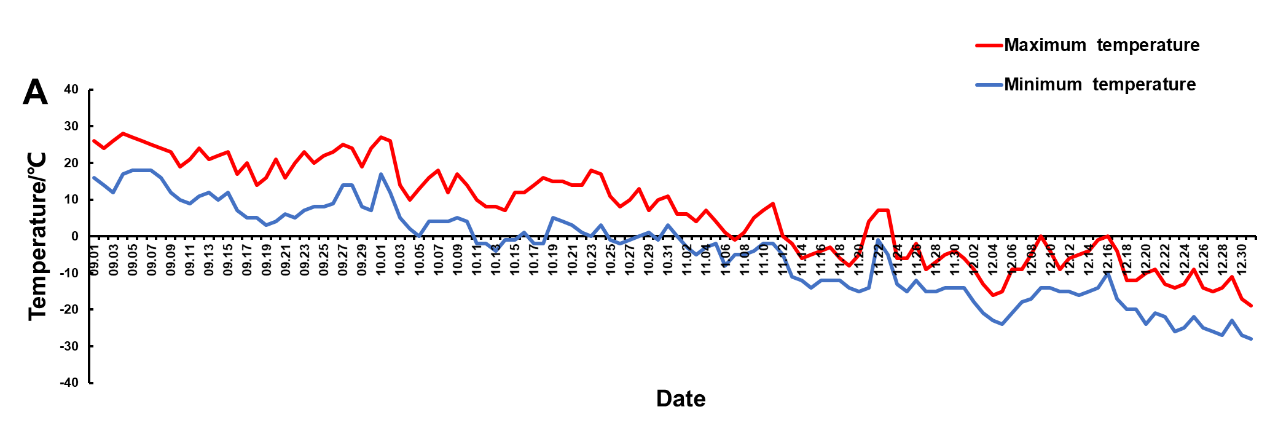


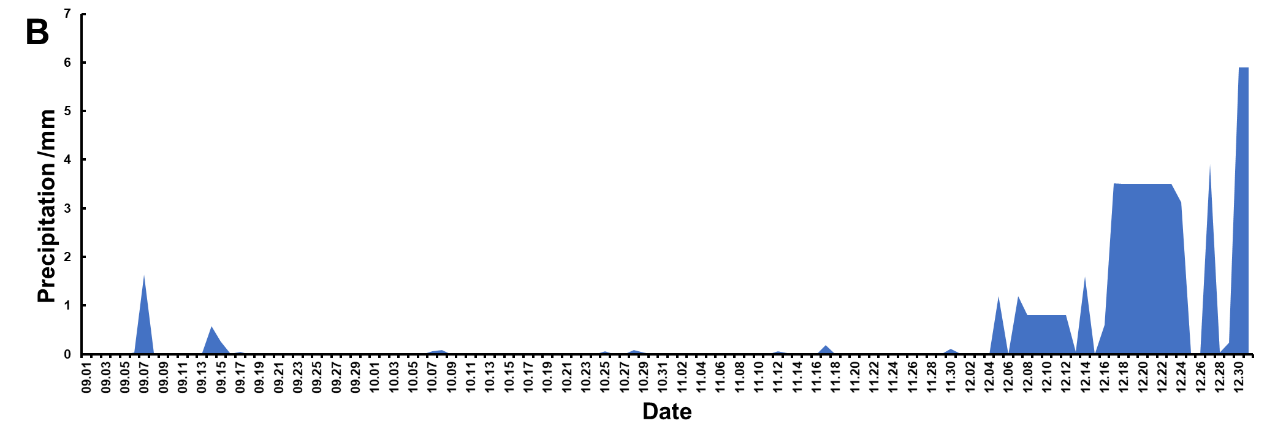


**Fig. S14** The field meteorological data during planting to completion of sampling. (A) Daily minimum and maximum temperatures (B) Daily precipitation
